# Integrated miRNA–mRNA network analysis identifies miR-182-5p as a potential regulator in COPD pathogenesis

**DOI:** 10.3389/fmed.2026.1743277

**Published:** 2026-06-22

**Authors:** Hannah Burke, Jodie Ackland, Bastian R. Angermann, Graham Belfield, Doriana Cellura, Damla Etal, Maria V. Humbert, Anna Freeman, Lisa Rydin, Kris Ostridge, Adam Platt, Alastair Watson, C. Mirella Spalluto, Karl J. Staples, Tom M. A. Wilkinson

**Affiliations:** 1 Faculty of Medicine, University of Southampton, Southampton, United Kingdom; 2 NIHR Southampton Biomedical Research Centre, University Hospital Southampton, Southampton, United Kingdom; 3 Translational Science and Experimental Medicine, Research and Early Development, Respiratory & Immunology, Biopharmaceuticals R&D, AstraZeneca, Gothenburg, Sweden; 4 Translational Genomics, Centre for Genomics Research, Discovery Sciences, Biopharmaceuticals R&D, AstraZeneca, Gothenburg, Sweden; 5 Early Clinical Development, Respiratory and Immunology R&D Biopharmaceuticals, AstraZeneca, Gothenburg, Sweden; 6 Translational Science and Experimental Medicine, Research and Early Development, Respiratory & Immunology, Biopharmaceuticals R&D, AstraZeneca, Cambridge, United Kingdom

**Keywords:** pulmonary disease, chronic obstructive, lung diseases, obstructive, extracellular vesicles, microRNAs, gene expression profiling, gene expression regulation, gene regulatory networks, miR-182-5p

## Abstract

**Introduction:**

Chronic obstructive pulmonary disease (COPD) is a leading cause of mortality worldwide and currently lacks effective disease-modifying therapies. Extracellular vesicles (EVs) are key mediators of intercellular communication and transport biologically active cargo, including microRNAs (miRNAs). We previously identified 8 differentially expressed EV miRNA (miR-223-3p, miR-2110, miR-182-5p, miR-200b-5p, miR-625-3p, miR-204-5p, miR-138-5p and miR-338-3p) that were differentially expressed in individuals with COPD compared with healthy volunteer ex-smoker controls (HV-ES). This study aimed to identify miRNA–mRNA interactions in diseased lung epithelium that may contribute to COPD pathogenesis.

**Methods:**

Gene expression was quantified by RNA sequencing of epithelial brushings obtained from 24 subjects with COPD and 20 HV-ES. *In silico* analyses were performed to identify target genes of the previously identified EV-derived miRNAs isolated from the same individuals. MiRNA–mRNA interactions were examined using negative correlation analysis and network-based approaches, and enrichment of biological processes was assessed using Cytoscape. Associations between computer tomography (CT) disease probability measures (DPM) and gene expression were assessed using Spearman correlation across the pooled cohort.

**Results:**

A total of 191 genes were differentially expressed in epithelial brushings from subjects with COPD compared with HV-ES. *In silico* analysis identified 121 miRNA–mRNA interactions involving these genes and the EV-associated miRNAs. Network analysis revealed miR-182-5p as a central hub, targeting multiple highly differentially expressed genes (DEGs). Expression of these DEGs correlated with CT DPM of emphysema and small airways disease (SAD). Exploratory pathway analyses suggested potential trends toward coordinated network regulation involving metabolic and immune –related processes; however, no biological processes remained significant after correction for multiple testing.

**Discussion:**

These findings highlight a potential role for EV-derived miRNA–mRNA regulatory networks in COPD pathogenesis, with miR-182-5p emerging as a putative regulator within this network. While exploratory analyses suggested possible associations with metabolic and immune-related pathways, these did not withstand multiple testing correction and should therefore be interpreted as hypothesis-generating. These findings support further mechanistic investigation of EV-derived miRNA–mRNA regulatory networks in COPD and may help inform future translational studies exploring their biological relevance.

## Introduction

Chronic obstructive pulmonary disease (COPD) morbidity and mortality continues to rise worldwide (1), in contrast to other chronic inflammatory diseases, where targeted molecular therapies have transformed clinical outcomes (2). COPD is characterized by persistent, dysregulated airway inflammation and progressive structural remodeling, driven by chronic exposure to noxious stimuli such as cigarette smoke. These insults initiate epithelial injury and immune activation leading to emphysema, small airways disease (SAD), and irreversible airflow limitation (3). Despite advances in symptomatic management, the lack of effective disease-modifying therapies reflects an incomplete understanding of the complex cellular and molecular networks that sustain inflammation and tissue damage in COPD.

The airway epithelium is a central orchestrator of COPD pathogenesis. Beyond its barrier function, epithelial cells act as active immune sentinels that sense environmental injury through pattern recognition receptors leading to activation of NF-κB and MAPK inflammatory cell signaling pathways. This results in the release of a broad array of cytokines including TNF-α, IL-1β and IL-6, chemokines and growth factors, which collectively drive the recruitment and activation of innate and adaptive immune cells (4). Neutrophils and macrophages accumulate within the airways and alveolar spaces, releasing proteases such as matrix metalloproteinases (MMPs) and reactive oxygen species, contributing to tissue destruction and emphysema (5). Adaptive immune responses, characterized by increased CD8^+^ cytotoxic T cells and B-cell–rich lymphoid follicles in the airway wall, further amplify inflammation through cytotoxic injury and antibody-mediated mechanisms (6).

Alveolar macrophages are the most abundant immune cells in the COPD lung and play a pivotal role in sustaining chronic inflammation. In COPD, macrophages exhibit altered polarization and impaired phagocytic capacity yet display exaggerated production of pro-inflammatory mediators. These responses are driven by aberrant activation of intracellular signaling pathways, perpetuating a feed-forward loop of epithelial–immune cell crosstalk (7). Importantly, both airway epithelial cells and macrophages demonstrate persistent transcriptional and epigenetic reprogramming in response to cigarette smoke exposure, suggesting that stable molecular regulatory mechanisms contribute to disease chronicity and heterogeneity (8).

MicroRNAs (miRNAs) have emerged as key post-transcriptional regulators of gene expression capable of coordinating these complex inflammatory networks. MiRNAs are small non-coding RNA molecules that that bind to complementary sequences within target mRNAs, leading to translational repression or mRNA degradation (9). A single miRNA can regulate hundreds of transcripts, while multiple miRNAs can converge on shared targets, enabling fine-tuned and context-dependent control of signaling pathways involved in inflammation, metabolism, apoptosis, and immune cell differentiation (10, 11). Dysregulation of miRNA expression has been implicated in multiple aspects of COPD pathobiology, including epithelial dysfunction, macrophage activation, oxidative stress responses, and aberrant immune signaling (12). Given their small size and conserved sequences, miRNAs represent attractive therapeutic targets, with both miRNA mimics and inhibitors currently under investigation in other inflammatory and fibrotic diseases (13).

However, miRNAs are inherently unstable in extracellular environments, and increasing evidence indicates that extracellular vesicles (EVs) serve as critical mediators of miRNA stability and intercellular transfer. EVs, including exosomes and microvesicles, are actively released by epithelial cells, macrophages, and other immune cells and carry selective molecular cargo comprising miRNAs, mRNAs, proteins, and lipids. Through uptake by recipient cells, EVs facilitate horizontal transfer of regulatory information and modulate inflammatory signaling, immune cell behavior, and tissue remodeling (14). In COPD, cigarette smoke has been shown to alter EV release and cargo composition, thereby reshaping intercellular communication within the lung microenvironment. Notably, Fujita et al. demonstrated that cigarette smoke extract–induced bronchial epithelial cell–derived EVs transport miR-210, which regulates autophagy and promotes myofibroblast differentiation in lung fibroblasts by targeting Atg7, linking EV-mediated miRNA signaling to airway remodeling (15).

Despite these advances, there remains a critical gap in understanding how EV-associated miRNAs derived directly from the COPD lung regulate gene expression within the airway epithelium itself. Using previously identified differentially expressed miRNA in EVs isolated from bronchoalveolar lavage fluid (BALF) in patients with COPD who were ex-smokers compared with healthy volunteer ex-smoker controls (HV-ES) (16), we sought to define their regulatory impact on epithelial gene expression in matched airway brushings. By integrating transcriptomic profiling with *in silico* miRNA–mRNA interaction analysis and network-based approaches, we aimed to identify key regulatory hubs and inflammatory pathways driving COPD pathogenesis. Elucidating these EV-mediated miRNA–mRNA networks may improve understanding of COPD pathobiology and help guide future mechanistic and translational studies of EV-mediated signaling.

## Materials and methods

### Study cohort and sample collection

**FIGURE 1:**
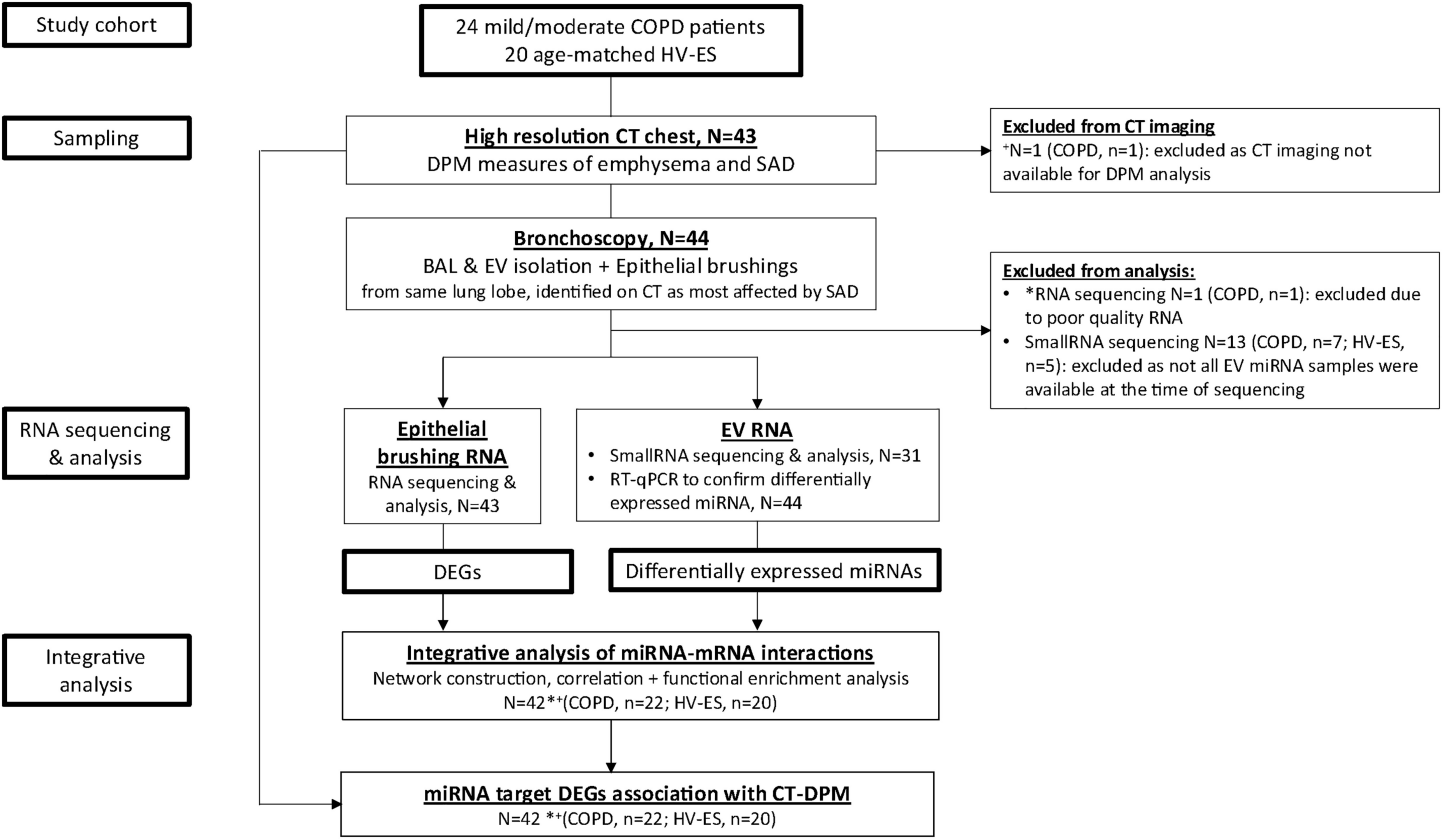
Subject enrolment and tests performed in the study to assess paired EV miRNA and epithelial brushing RNA expression and analysis. BAL, bronchoalveolar lavage; COPD, chronic obstructive pulmonary disease; CT, computer tomography; DEGs, differentially expressed genes; DPM, disease probability measures; EV, extracellular vesicle; HV-ES, healthy volunteer ex-smoker controls; miRNA, microRNA; RT-qPCR, real-time quantitative PCR; SAD, small airways disease. Figure created in PowerPoint, MS Office 365.

The MICAII study recruited subjects with stable, mild or moderate COPD as defined by Global initiative for Chronic Obstructive Lung Disease (GOLD) guidelines (17), alongside HV-ES. All subjects had >10 pack year history and had stopped smoking at least 6 months prior to study enrolment. Exclusion criteria included a history of other pulmonary disease, α-1- antitrypsin deficiency, long-term antibiotics/oral steroids, or an exacerbation within the month prior to recruitment. All MICAII study subjects with recovered BALF suitable for EV isolation were included, resulting in twenty-four subjects with mild or moderate COPD and 20 HV-ES (demographics given in Table 1). All subjects gave written informed consent, including consent for linkage of samples to clinical data. The MICAII study was approved by and performed in accordance with National Research Ethics Service South Central ethical standards - Hampshire A and Oxford C Committees (LREC no: 15/SC/0528) (16, 18–22). As previously described, subjects underwent volumetric computer tomography (CT) chest scans in full inspiration and maximum expiration using a Siemens Sensation 64 scanner (20). Low attenuation area below −950 Hounsfield Units (%LAA) was calculated as a disease probability measure (DPM) of emphysema, and prebronchodilator, single-breath diffusion was performed, as per guidelines, with percent predicted carbon monoxide transfer coefficient calculated (DLCO%). A DPM for SAD was measured using the ratio of mean lung attenuation on expiratory and inspiratory scans (E/I MLD). Of the subjects included in this study, one volunteer with COPD did not undergo CT imaging for DPM of emphysema and SAD to be calculated. Subject numbers, sample processing, and subsequent analysis are summarized in Figure 1.

**TABLE 1:** Characteristics of the study cohort, *N* = 44.

Subject characteristics	COPD (*n* = 24)	HV-ES (*n* = 20)	Test statistic	*P*-value
Age, mean ± SD	70.1 ± 6.9	68 ± 7.3	*t* = 0.97	0.34
Male, *n* (%)	20 (83)	11 (55)		0.06
Smoking pack years, median (IQR)	50.0 (20–63.8)	25.0 (19–38.8)	*W* = 331	**0.034**
BMI, mean ± SD	29.6 ± 4	28.4 ± 4	*t* = 1.1	0.3
FEV1 (% predicted), mean ± SD	77.2 ± 13.6	101.5 ± 14.1	*t* = −5.8	**<0.00001**
FVC (% predicted), mean ± SD	100.0 ± 14.6	101.2 ± 16.2	*t* = −0.25	0.65
FEV1/FVC%, mean ± SD	59.0 ± 8.4	77.5 ± 4.6	*t* = −9.2	**<0.00001**
FEF 25–75 (% predicted), mean ± SD	44.0 ± 17.8	103.4 ± 24.8	*t* = −9.0	**<0.000001**
DLCO (% predicted), mean ± SD	75.0 ± 14.3	84.4 ± 13.2	*t* = −3.1	**0.004**
SAD DPM*, median (IQR)	25.7 (18.6–33.8)	16.0 (12.2–19.17)	*W* = 373	**<0.001**
Emphysema DPM*, median (IQR)	9.0 (3.6–19.1)	1.6 (0.9–4.1)	*W* = 368	**<0.001** 0.41
COPD status, GOLD stage, *n* (%)				
Mild	10 (42)	NA		
Moderate	14 (58)	NA		

Fisher’s exact test was performed for gender given small sample size. Chi-squared test used for COPD status. Shapiro-Wilk test for normality was performed for all continuous variables. Welch two-sample *t*-test was performed for normally distributed data; Age, BMI, FEV1, FVC, FEV1/FVC and FEF 25–75, DLCO. Wilcoxon rank sum test was performed for skewed data; smoking pack years, SAD DPM and Emphysema DPM. BMI, body mass index; DPM, disease probability measures; FEV1, forced expiratory volume in 1 s, FVC, forced vital capacity; FEF, forced expiratory flow rate; DLCO, diffusion capacity of the lung for carbon monoxide; HV-ES, healthy volunteer ex-smoker controls IQR, interquartile range; NA, non-applicable; SAD, small airways disease; SD, standard deviation. *Data only available for *n* = 23 COPD patients. *P*-values shown in bold are significant.

### EV isolation and miRNA sequencing

Bronchoalveolar lavage (BAL) and epithelial brushings were performed from the same lung lobe location, identified as the lobe with the most SAD by CT measurement, in each subject as previously described (16, 21, 22). BAL was performed by instilling 100 mL of pre-warmed 0.9% saline in 20 mL aliquots, followed by gentle aspiration. As CT imaging was unavailable for one subject with COPD, the right middle lobe was sampled, as this was the most frequent site of SAD among the other COPD subjects.

Bronchoalveolar lavage fluid was poured through 100 μm cell strainer to remove mucus and cells were removed by centrifugation at 400 *g*, 4 *^o^*C for 10 min. The cell-free supernatant was stored at −80 *^o^*C prior to EV isolation (see Supplementary methods for processing of the cell pellet). Fifteen milliliters of BALF were centrifuged at 400 *g* for 10 min to remove cell debris, filtered through a 0.22 μm PVDF, 33 mm gamma sterilized filter (Merck Millipore^®^, Watford, UK) to remove larger particles (e.g., apoptotic bodies), and then concentrated using a Amicon^®^ Ultra-15 (10,000 MWCO) spin filter according to manufacturer’s instructions. The resultant ∼2 mL EV containing sample passed through a size exclusion chromatography platform (PURE-EV™, HansBioMed^®^, Tallinn, Estonia) under gravity flow with the addition of 10 mL of 1× phosphate-buffered saline (PBS). Twenty-four 500 μL fractions were collected, and fractions 6–11 were pooled as the EV-enriched fraction.

To ensure consistency across samples, EVs were isolated from a fixed starting volume of BALF (15 mL) for all participants. This approach was selected to minimize technical variability and maintain biological comparability between groups.

A subset of EVs (*n* = 9) underwent characterization. EV-associated protein was quantified using a CD9 double sandwich enzyme-linked immunosorbent assay (CD9 ELISA, ExoTest™, HansBioMed^®^), performed according to the manufacturer’s instructions. Transmission electron microscopy with negative staining was used to confirm vesicular morphology and size. SDS-PAGE and Western blotting were performed to assess EV marker enrichment and cellular contamination. Briefly, EVs were lysed and denatured in NuPAGE™ LDS Sample buffer (4×) with 12% 2-mercaptoethanol (ThermoFisher Scientific^®^) at 70 °C for 10 min and resolved on NuPAGE™ 4%–12% Bis-Tris protein gels (ThermoFisher Scientific^®^). Membranes were probed for CD63 (anti-rabbit polyclonal antibody, Atlas Antibodies, Bromma, Sweden) and the endoplasmic reticulum marker calnexin (anti-rabbit monoclonal antibody, Cell Signaling Technology, Danvers, US), demonstrating enrichment of EV-associated proteins and absence of intracellular contaminants (16).

Nanoparticle tracking analysis (NTA) or equivalent particle sizing and concentration measurements were not performed due to limited sample availability and lack of instrumentation. We acknowledge that this precludes direct quantification of EV particle number and size distribution and limits interpretation of whether observed differences in EV-associated miRNA expression reflect altered miRNA cargo composition, differences in EV abundance between individuals or groups, or a combination of both. Given the ongoing lack of consensus regarding optimal EV normalization strategies, and the potential for particle-based normalization to obscure biologically relevant differences in EV yield, a fixed input volume approach was used. This limitation and its implications are further discussed in the section “Discussion.”

As previously described, prior to RNA isolation, BAL EVs (suspended in 200 μL of 1× PBS) were lysed using Buffer RPL at room temperature for 3 min. Total RNA was extracted from BAL EVs using the miRNeasy Serum/Plasma Advanced kit (Qiagen^®^) with Qiaseq miRNA Library Quality Control (QC) spike-ins to monitor extraction quality, showing excellent consistency across samples (R^2^ = 0.94–1.0). Small RNA sequencing libraries were prepared using the Qiaseq miRNA Library Kit (Qiagen), assessed with an Agilent Bioanalyzer 2100, and sequenced on an Illumina NextSeq500 (Illumina^®^, Chesterford, UK). Samples were balanced across sequencing batches by disease/control status. Reads were quality-checked with *FastQC* (v0.11.7), trimmed with *cutadapt* (v1.11), and aligned to the human genome (GRCh37/hg19) and *miRBase* (v_20) using *bowtie2* (v2.2.2), with an average6 genome-mapping rate of 53.4% (16) (see Supplementary methods for further detailed methods).

### Small RNA sequencing analysis and validation with RT-qPCR

Prior bioinformatic and statistical analysis of small RNA sequence data was previously performed and is reported in detail in (16), where 8 significantly differentially expressed lung EV miRNAs between HV-ES and COPD were identified.

Briefly, the small RNA sequencing analysis was performed with R (v3.6.2). Prior to filtering, 2308 miRNA were detected across all samples. All filtering was performed on log-transformed counts per million (CPM) data. Lowly expressed miRNAs were filtered out, using a cut-off of >10 CPM in a minimum of 15 samples (see Supplementary methods for validation). The filtered miRNA dataset was TMM normalized, differential expression analysis was performed using the *edgeR* (v3.14.0) exact test for two-group comparisons (HV-ES and COPD) under a negative binomial model, incorporating common, trended, and tagwise dispersion estimate and corrected for multiple testing using the Benjamini-Hochberg (BH) false discovery rate (FDR). FDR adjusted *p*-values < 0.05 were considered statistically significant.

Identified EV miRNA targets from the small RNA sequencing were validated using RT-qPCR in all 24 patients with COPD and 20 HV-ES. Detailed methodology can be found in the Supplementary methods.

### RNA isolation and sequencing

RNA was isolated from epithelial brushings and processed for RNA sequencing as previously described (22). Total RNA was extracted from epithelial brushings using the AllPrep DNA/RNA/miRNA Universal Kit (Qiagen^®^). The quantity and quality of RNA samples were determined using the standard RNA analyser kit on a 96-channel Fragment analyser (Agilent^®^ Technologies, Santa Clara, US). Extracted samples with a yield concentration >25 ng/μl total RNA, and a DV200 value (percentage of RNA fragments > 200nucleotides) ≥30% were deemed to be of sufficient quantity and quality for Total RNA-seq analysis. Samples were diluted to 25 ng/μl using a Tecan Fluent liquid handling automation system (Tecan, Männedorf, Switzerland). Library preparation was done in four separate runs, one 96 well plate per run. The Kapa RNA HyperPrep Kit with RiboErase (HMR) was used for reverse transcription, generation of double stranded cDNA and subsequent library preparation and indexing to facilitate multiplexing (Roche, Basel Switzerland), all of which was performed through automation on a Tecan fluent. The libraries were quantified with the 96-channel Fragment Analyzer using the standard sensitivity next generation sequencing (1) kit (Agilent^®^ Technologies). Samples from each preparation plate were pooled and the final pools (4 in total) were quantified using a Qubit instrument for concentration determination with the DNA High Sensitivity kit (ThermoFisher Scientific). Fragment size was determined using the Fragment Analyzer, standard sensitivity NGS kit (Agilent^®^ Technologies). Three of four library pools were further diluted to 1 nM and sequenced on a NovaSeq 6000 (Illumina^®^) using NovaSeq 6000 S4 Reagent Kit, 2 × 76 cycles. The remaining library pool was diluted to 1.9 nM and sequenced on NovaSeq 6000 (Illumina^®^) using 2 NovaSeq 6000 SP S1 Reagent Kits, 2 × 51 cyclers. Average reads per sample were 52.6 million.

FASTQ files generated from 44 epithelial brushings were collected and read quality for all libraries was assessed using *FastQC* tool (v0.11.9) (23), *Qualimap* (v2.2.2d) (24) and *samtools* stats (v1.15) (25). QC metrics for *Qualimap* were based on a STAR (v2.7.10a) (26) alignment against the human genome (GRCh38, Gencode v43). Next, QC metrics were summarized using *MultiQC* (v1.12) (27). Sequencing adapters were then trimmed from the remaining libraries using *NGmerge* (v0.3) (28). A human transcriptome index consisting of cDNA and ncRNA entries from Gencode (v43) was generated and reads were mapped to the index using *Salmon* (v1.7.0) (29). Estimated counts from *Salmon* were used as input for *DESeq2* (v1.34.0) using *tximport* (v1.22.0) (30). The bioinformatics workflow was organized using *Nextflow* workflow management system (v20.10) (31) and *Bioconda* software management tool (32).

### RNA sequencing analysis

RNA Sequencing data from epithelial brushings, matched to the same subjects used for small RNA sequencing, were analyzed to determine differentially expressed mRNA between HV-ES and COPD subjects using *DESeq2* (v1.34.0) (33) and “ashr” (v2.2_54) (34) for fold change (FC) shrinkage using “lfcShrink.” In the models used to assess differential expression between HV-ES and COPD groups, effects from gender and a technical batch-effect (library prep plate) were incorporated into the model design as covariates. Genes were determined to be differentially expressed using an adjusted *p*-value < 0.05 calculated using the BH multiple testing correction method. Visualization of gene expression were performed using the *EnhancedVolcano* package (v1.20.0).

### Integrative analysis of miRNA–mRNA interactions

To explore and describe the interactions between the eight previously identified differentially expressed lung EV miRNA and mRNA from matched epithelial brushings, we applied an integrated bioinformatic workflow combining target prediction, correlation analysis, and network-based approaches (summarized as a workflow in the Supplementary methods and described in detail below).

### miRNA target identification and definition of network edges

Validated and predicted targets of our eight differentially expressed miRNA were identified using the *multiMiR* package (v1.24) (35) in R (v4.3.2). We queried all experimentally validated interaction databases available within *multiMiR* (v1.24) (miRecords, miRTarBase, and TarBase) and all prediction databases (DIANA-microT, ElMMo, MicroCosm, miRanda, miRDB, PicTar, PITA and TargetScan). To reduce false-positive predictions, filtering was restricted to (i) all experimentally validated targets and (ii) the top 20% of predicted targets ranked by database-specific confidence scores.

This initial query yielded >21,000 potential targets across the eight differentially expressed EV miRNAs. To refine the interaction set to biologically relevant candidates in our dataset, we intersected these targets with the list of 191 differentially expressed genes (DEGs) identified in epithelial brushings. Furthermore, consistent with canonical miRNA-mediated repression, we required inverse differential expression for candidate interactions (i.e., significantly upregulated miRNAs were paired only with significantly downregulated DEGs in COPD, and vice versa).

### miRNA-mRNA correlation analysis

To assess expression-level support for predicted or validated target interactions, Pearson’s correlation coefficients were calculated between log2 normalized miRNA and mRNA expression values using the “*rcorr*” function in the *Hmisc* package (v5.1.1) in R (v4.3.2) across all subjects (COPD and HV-ES combined). *P*-values were adjusted for multiple testing using the BH procedure using the “p.adjust” function from the base R *stats* package (v4.3.2), and an FDR-adjusted *p*-value < 0.05 was considered statistically significant. Significant negative correlations were used as an additional attribute of network *nodes* but were not mandatory for *edge* inclusion (see below).

### miRNA-mRNA network construction and analysis

Network *edges* were defined as miRNA–mRNA pairs that met all the following criteria in a single integrated framework:

1. Identified as a validated target (from miRecords, miRTarBase, or TarBase) or as a top 20% confidence predicted target (from DIANA-microT, ElMMo, MicroCosm, miRanda, miRDB, PicTar, PITA or TargetScan) using *multiMiR* (v1.24).
2. Present within the list of differentially expressed epithelial genes; and
3. Demonstrated inverse differential expression between miRNA and mRNA in COPD.

Significant negative Pearson correlation (FDR < 0.05) was not required for *edge* inclusion but was incorporated as a *node*-level annotation to indicate additional expression-based support.

This multiMiR-derived, inverse-expression-filtered *edge* set was used for all downstream network analyses, including degree centrality calculations, identification of dominant miRNAs, assessment of miRNA synergism, and enrichment analyses.

Network topology and centrality metrics were analyzed using the default settings of miRmapper (36) functions including “*getImpact*” to identify the predicted impact of each miRNA on the DEGs. Networks were visualized in *Cytoscape* (v3.10.1), integrating node attributes from previously generated outputs such as differential expression magnitude and correlation significance (37). Sub-cluster structure within the miRNA–mRNA network was assessed using the *EAGLE* algorithm implemented in the *ClusterViz* (v1.0.3) plugin (38) using parameters CliqueSize Threshold = 3 and ComplexSize Threshold = 2. The built in *Cytoscape* (v3.10.1) *Network Analyser* tool was used to assess additional network metrics including Degree, Betweenness Centrality, Closeness Centrality, and Stress.

### Functional enrichment analysis

The Biological Networks Gene Ontology tool (*BiNGO* v3.0.5) (39) was used within *Cytoscape* (v3.10.1) to analyze functional enrichment of miRNA-mRNA networks. Analyses were performed using the Gene Ontology (GO) Biological Processes (BP), with the whole annotation set as the reference, the hypergeometric test for statistical significance, and BH correction for multiple hypothesis testing using default settings. The *EnrichmentMap* (v3.4) and *AutoAnnotate* (v1.5.1) plugins (40) within *Cytoscape* (v3.10.1) were subsequently used to organize and display the major enriched functional themes from the GO enrichment results. Enrichment results were visualized using the *EnrichmentMap* plugin in *Cytoscape* (v3.10.1), where nodes represent GO:BP terms. Gene set similarity was calculated using the Jaccard coefficient with a cutoff of 0.35 to define edges between GO:BP terms. Gene set filtering was performed using a *p*-value threshold of 0.01, and all resulting nodes were retained for downstream analysis. Network annotation was performed using the *AutoAnnotate* plugin (v1.5.1). Cluster labels were generated from GO:BP term names using the WordCloud app, with a maximum of three words per label, a minimum word occurrence of one, and an adjacent word bonus of eight. Labels were subsequently manually curated for readability. All significant GO:BP nodes, including singletons, were retained in the final network representation.

### Statistical analysis

Demographic and baseline characteristics of COPD subjects and HV-ES were analyzed using SPSS (v27) and GraphPad^®^ Prism (v10.0). Categorical variables were compared using the Chi-squared test (for counts > 5) or Fisher’s exact test (for counts ≤ 5). The Shapiro–Wilk test was used to assess the normality of continuous variables. Depending on distribution, continuous variables were compared using Welch’s two-sample *t*-test (normal distribution) or the Mann–Whitney U test (non-normal distribution).

Paired CT-derived DPM for SAD and emphysema, together with RNA-sequencing data from epithelial brushings, were available for forty-two subjects (22 COPD and 20 HV-ES). However, because CT abnormalities differed between COPD and HV-ES subjects, these pooled analyses should be interpreted cautiously, as observed differences may in part reflect diagnostic group differences. Given the non-parametric distribution of the CT-derived measures, associations between DPM and log2-normalized DEG expression were assessed using Spearman’s rank correlation, implemented via the “*rcorr*” function in the *Hmisc* package (v5.1.1) in R (v4.3.2). Correlations were visualized using *corrplot* (v0.92), with asterisks indicating significance after adjustment for multiple testing using the BH method using the base R *stats* package (v4.3.2) (*p < 0.05, **p < 0.01).

## Results

### Study cohort characteristics

The clinical characteristics of twenty-four patients with COPD and twenty HV-ES are shown in Table 1. The subjects were matched for age, sex, and body mass index (BMI). Although groups were matched for smoking history, COPD patients had significantly higher pack-years compared with HV-ES (Wilcoxon rank-sum test, *W* = 331, *p* = 0.034). As expected, disease defining characteristics such as forced expiratory volume in 1 s (FEV1) % predicted, FEV1/Forced vital capacity (FVC), forced expiratory flow rate (FEF) 25%–75% predicted and diffusion capacity of the lung for carbon monoxide (DLCO) % predicted were significantly reduced in the COPD group. CT-derived measures of SAD and emphysema were also significantly elevated in the patients with COPD. The COPD subjects varied from mild to moderate disease, with a mean FEV1% predicted of 77.5% (SD ± 14.8).

### Differentially expressed BAL EV miRNA and epithelial brushing genes in paired samples in COPD compared with HV-ES

Our previous published analysis revealed five upregulated miRNA (miR-223-3p, miR-2110, miR-182-5p, miR-200b-5p and miR-625-3p) and three downregulated miRNA (miR-204-5p, miR-138-5p and miR-338-3p) in patients with COPD compared with HV-ES (16), (summarized in Supplementary Figures 1, 2 and Supplementary Table 1). MiR-223-3p had the highest FC, whereas miR-204-5p had the lowest negative FC in COPD. Of note, miR-625-3p was only detected in the BAL EVs of 18 (75%) patients with COPD and 12 (60%) HV-ES.

**FIGURE 2:**
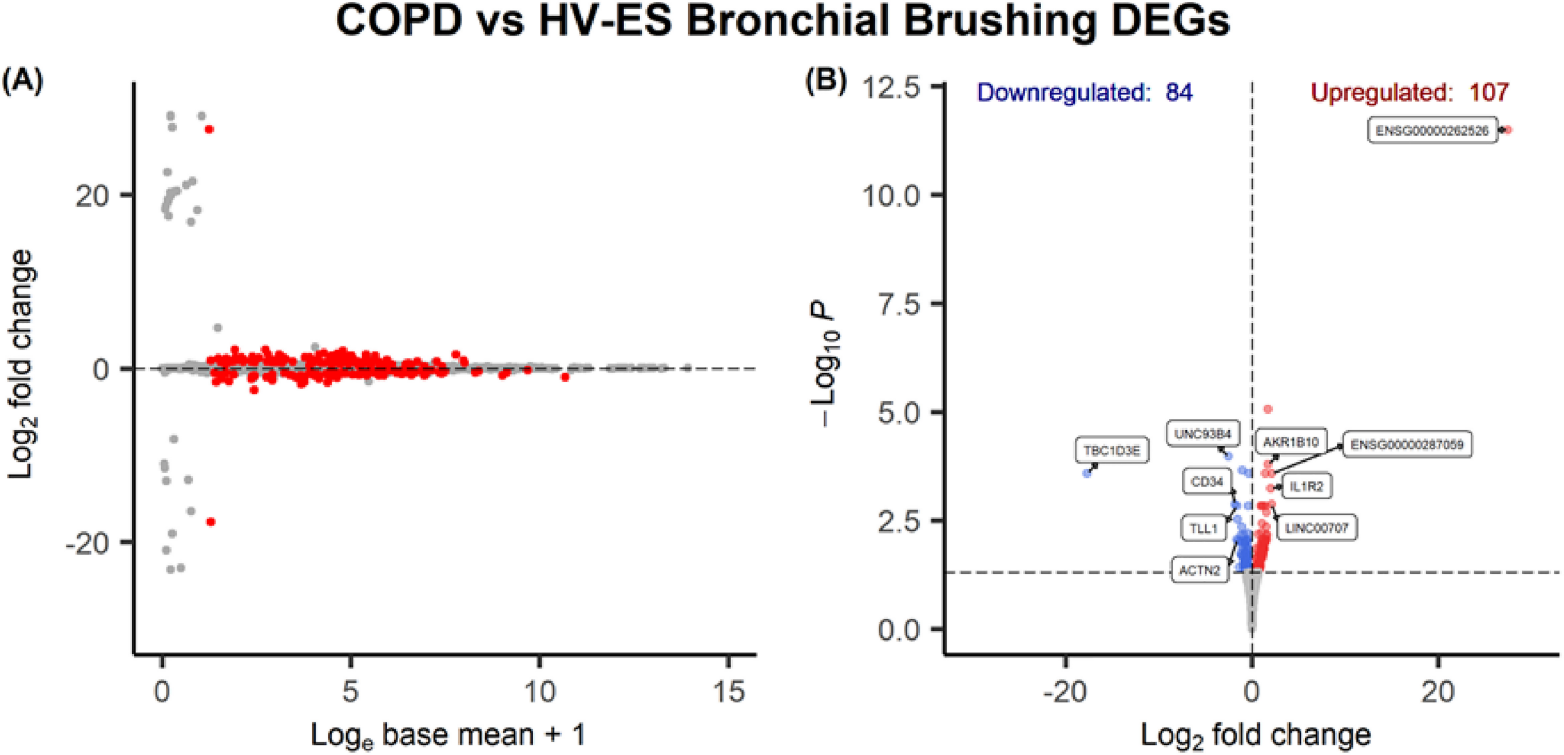
Differentially expressed mRNA (*n* = 191) in bronchial epithelial brushings between COPD subjects and HV-ES. **(A)** MA plot illustrating the relationship between mean expression and log fold change. Red dots show genes with *P*-values < 0.05 after FDR correction for multiple testing. **(B)** Volcano plot displaying the significance (adjusted *p*-value) versus logFC for DEGs between COPD and HV-ES samples, with upregulated (*n* = 107) genes in red and downregulated (*n* = 84) genes in blue. The top 5 significantly dysregulated genes are labeled. Horizontal dashed line indicates FDR *p*-value 0.05. COPD, chronic obstructive pulmonary disease; DEG, differentially expressed genes; FC, fold change; FDR, false discovery rate; HV-ES, healthy volunteer ex-smoker controls. Figure created using EnhancedVolcano (v1.20.0) in R (v4.3.2).

In this study, differential gene expression in paired epithelial brushing samples taken from the same lung lobe location as the BAL EV sample demonstrated 191 DEGs between groups with an FDR < 0.05 (Figure 2A and Supplementary Table 2). Of the 191 DEGs, there were 39 genes with a log2FC of greater than one (most upregulated in COPD) and 16 genes with a log2FC less than minus one (most downregulated in COPD). The top five up- and downregulated genes in the COPD epithelial brushings are labeled in Figure 2B.

### Identification of key COPD associated EV miRNA-mRNA targets

**FIGURE 3:**
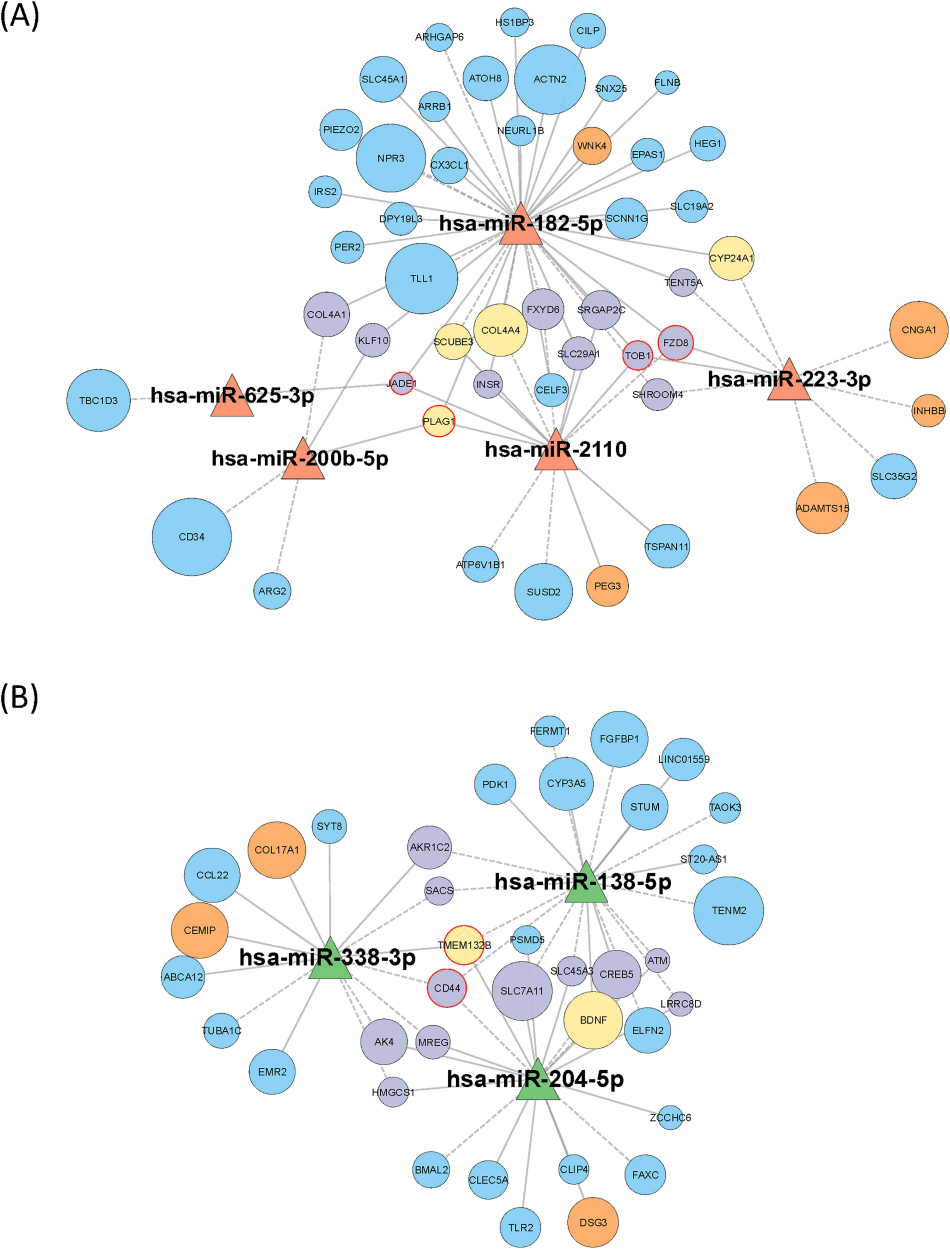
miRNA-mRNA interaction networks for **(A)** upregulated and **(B)** downregulated EV miRNA and corresponding DEGs in epithelial brushings from COPD patients. **(A)** Red triangular nodes represent upregulated EV miRNAs in COPD. Circular nodes represent DEGs that are downregulated in COPD and inversely paired with these miRNAs. **(B)** Green triangular nodes represent downregulated EV miRNAs in COPD. Circular nodes represent DEGS that are upregulated in COPD and inversely paired with these miRNAs. **(A,B)** Edges represent miRNA–mRNA target relationships identified using *multiMiR* (v1.24) after filtering for experimentally validated targets and the top 20% confidence predicted targets, restricted to DEGs showing inverse regulation. Solid edges indicate experimentally validated targets; dotted edges indicate predicted targets. Circular node color reflects additional network attributes: Purple - degree centrality > 1 (targeted by more than one miRNA); Orange - significant negative miRNA–mRNA correlation (Pearson’s correlation, FDR < 0.05); Yellow - both degree centrality > 1 and significant negative correlation, and Blue: neither attribute. Circular node size is proportional to the magnitude of differential expression in COPD [larger nodes indicate greater down-regulation in panel **(A)** or greater up-regulation in panel **(B)**]. Circular nodes outlined with a red border denote those with the highest degree centrality in the network. COPD, chronic obstructive pulmonary disease; DEG, differentially expressed genes; EV, extracellular vesicles; FDR, false discovery rate; miRNA, microRNA; RT-qPCR, real-time quantitative PCR. Figure created in *Cytoscape* (v3.10.1).

To explore regulatory interactions between EV miRNAs and epithelial gene expression in COPD, we integrated the eight dysregulated EV miRNAs with epithelial mRNA differential expression data using the multiMiR database. In the network (Figure 3), *edges* represent miRNA–mRNA target relationships (solid lines = experimentally validated targets; dotted lines = predicted targets), while *nodes* represent DEGs from epithelial brushings. *Node* size is proportional to the magnitude of differential expression in COPD (i.e., larger nodes reflect greater downregulation in panel A or upregulation in panel B), whilst *node* color is described below.

Overall, 121 miRNA-mRNA interactions (*edges*) were identified, with 87 (46%) DEGs targeted by at least one dysregulated miRNA. Among the five upregulated EV miRNAs (Figure 3A), 49 unique epithelial gene targets were identified, of which 43 interactions had prior experimental validation (solid *edges*). Fifteen target genes (*FZD8*, *JADE1*, *TOB1*, *PLAG1*, *CYP24A1*, *COL4A4*, *TENT5A*, *FXYD6*, *SCUBE3*, *SHROOM4*, *COL4A1*, *SLC29A1*, *KLF10*, *INSR* and *SRGAP2C*) demonstrated a degree centrality > 1 (i.e., targeted by more than one miRNA) and are depicted as purple or yellow *nodes* in Figure 3A (purple = degree centrality > 1; yellow = both degree centrality > 1 and significant correlation – see below), indicating potential coordinated or synergistic regulation.

For the downregulated miRNA (Figure 3B), thirty-eight unique gene targets were identified, with 24 experimentally validated interactions (solid *edges*). Thirteen target genes (*TMEM132B*, *CD44*, *HMGCS1*, *MREG*, *CREB5*, *ATM*, *SLC7A11*, *AKR1C2*, *SLC45A3*, *AK4*, *LRRC8D*, *BDNF* and *SACS*) also showed degree centrality > 1 and are depicted as purple or yellow *nodes* in Figure 3B.

Correlation analysis between EV miRNA expression and matched epithelial mRNA levels across COPD and HV-ES subjects identified 114 significant negative miRNA–mRNA correlations (FDR < 0.05). Of these, 15 overlapped with experimentally validated or predicted targets identified by multiMiR and are represented in Figure 3 as orange or yellow *nodes* (orange = significant correlation only; yellow = both significant correlation and degree centrality > 1). Six genes–*PLAG1*, *CYP24A1*, *COL4A4*, and *SCUBE3* (Figure 3A), and *TMEM132B* and *BDNF* (Figure 3B)–met both criteria and are therefore highlighted as yellow *nodes*, supporting coordinated multi-miRNA regulation. The outputs from the multiMiR analysis and the correlation results are summarized in Supplementary Table 3 and the Supplementary data file: multiMiR analysis and correlation results for up- and downregulated miRNAs.

Network topology analysis identified six genes with the highest degree centrality (red-bordered *nodes*), indicating extensive miRNA connectivity. Notably, all three downregulated miRNAs (miR-138-5p, miR-204-5p, and miR-338-3p) targeted the upregulated genes *TMEM132B* and *CD44*, which showed the highest degree centrality (Figure 3B). In contrast, four downregulated genes (*FZD8*, *JADE1*, *TOB1* and *PLAG1*) were each targeted by different upregulated miRNAs. However, miR-2110 and miR-182-5p shared several mRNA targets, including multiple central *nodes*, suggesting that they may act as synergistic regulators within the COPD miRNA–mRNA interaction network (Figure 3A).

### miR-182-5p has the highest centrality in the miRNA-mRNA interaction network

To evaluate the potential regulatory influence of individual miRNAs within the interaction network, we examined their predicted impact on epithelial target genes. The differential expression of miR-223-3p and miR-182-5p in COPD EVs was established in our previous small RNA-sequencing study (log2FC = 2.97, FDR = 0.02 and log2FC = 1.52, FDR = 0.04, respectively) (16). Here, we build upon those findings by assessing their predicted regulatory interactions with epithelial differentially expressed genes within the constructed miRNA–mRNA network.

Although miR-223-3p was the most upregulated miRNA in EVs in COPD, our current network analysis suggests that it is predicted to influence only a limited subset of target genes, affecting 4.71% of all the DEGs and 10.34% of all genes targeted by miRNAs in the dataset (Supplementary Table 4). In contrast, miR-182-5p–despite a smaller FC –was associated with the largest number of targets in this integrated analysis, influencing 19.9% of DEGs and 43.68% of all miRNA-targeted genes (Supplementary Table 4). Notably, several of these predicted target’s rank among the most strongly downregulated genes in epithelial brushings, including *TLL1* (log2FC −1.6, *p* = 0.001), *ACTN2* (log2FC −1.59, *p* = 0.009), *NPR3* (log2FC −1.55, *p* = 0.003) and *COL4A4* (log2FC −1.075, *p* = 0.02) (Supplementary Table 2).

To further characterize network topology, we next performed network clustering analysis to identify interconnected subclusters that could represent candidate functional modules. Six clusters were identified (Supplementary Figure 3). Cluster 1 included miR-204-5p and miR-138-5p, while Cluster 3 included miR-2110 and miR-625-3p and the remaining miRNAs were assigned to individual clusters. Cluster 2, which includes miR-182-5p, exhibited the highest out-degree score, suggesting that this cluster–and particularly miR-182-5p–has a high degree of connectivity within the miRNA–mRNA network.

To assess the robustness of miR-182-5p centrality, out-degree was evaluated across validated targets only and predicted targets at thresholds ranging between 5% and 30%. miR-182-5p consistently exhibited the highest connectivity, regardless of threshold or target type (Supplementary Figures 4A,B). Additional centrality metrics generated using the 20% predicted target threshold (Supplementary Figures 4C–F) confirmed that miR-182-5p ranks highest across Degree, Betweenness Centrality, Closeness Centrality, and Stress network measures and these rankings were stable across all metrics (Supplementary Figure 4G), supporting a putative role for miR-182-5p as a key regulator within this miRNA–mRNA network. While these findings highlight miR-182-5p and other highly connected miRNAs as putative or candidate regulators, we emphasize that network centrality is hypothesis-generating and constrained to the context of this experimental design and does not alone provide definitive evidence of biological dominance. These observations provide a framework for prioritizing miRNAs for future experimental validation rather than conclusive functional inference.

### GO enrichment of biological processes and pathways in miRNA-mRNA interactions in COPD

To explore potential biological pathways associated with the eight key candidate COPD EV miRNAs in disease pathogenesis, we performed GO enrichment analysis on the 87 unique genes in the miRNA-mRNA interaction network. This analysis was intended to be exploratory and hypothesis-generating.

Although 88 GO biological process terms reached nominal significance (*p* < 0.01), none remained significant after correction for multiple testing (FDR < 0.05) (Supplementary Table 5). Given the modest sample size and the relatively small gene set derived from the filtered interaction network, these findings should be interpreted cautiously and are not presented as definitive evidence of pathway enrichment.

**FIGURE 4:**
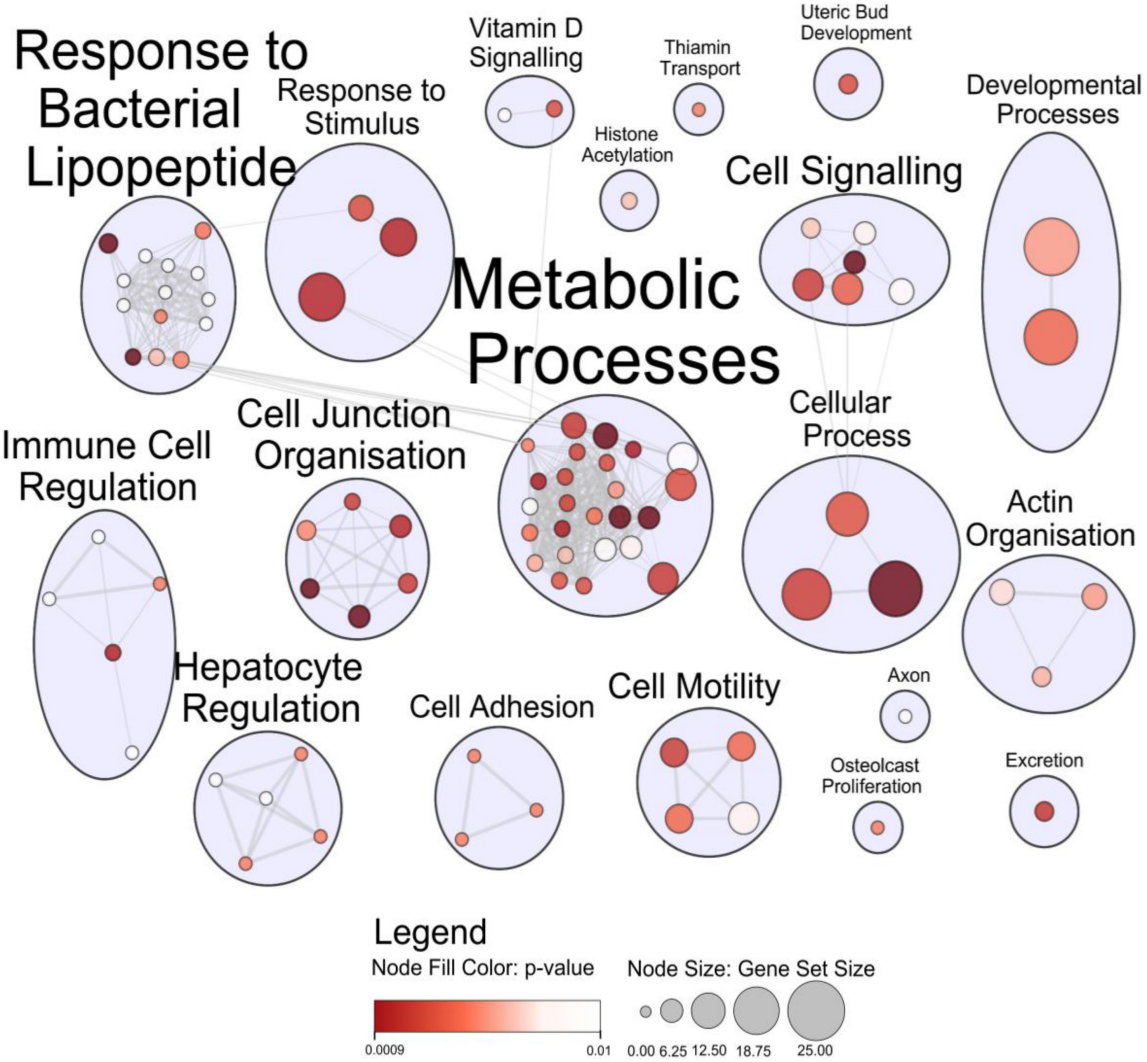
Enrichment clusters show key processes associated with mRNA dysregulated in COPD. Enrichment analysis was performed using BiNGO in *Cytoscape* (v3.10.1) on the mRNAs identified to have a predicted or validated interaction with at least one dysregulated miRNA. Terms were considered statistically significant if *p* < 0.01 using the Hypergeometric test. The enrichment output (Supplementary Table 5) was summarized using EnrichmentMap (v3.4) and AutoAnnotate (v1.5.1) to cluster significantly enriched GO Biological Process (BP) terms (*p* < 0.01) based on semantic similarity using a Jaccard score of 0.35. Nodes represent individual GO:BP terms, colored by enrichment significance and size according to the number of genes assigned to each term. Edges between nodes indicate gene overlap between terms, with thicker edges representing a greater number of shared genes. DEG, differentially expressed genes; FDR, false discovery rate; miRNA, microRNA; GO:BP, Gene Ontology: Biological Process. Figure created in *Cytoscape* (v3.10.1).

For descriptive purposes, enriched GO terms were visualized using EnrichmentMap and summarized into broader biological themes using AutoAnnotate (Figure 4). The largest cluster comprised terms related to metabolic processes, which were among the most nominally enriched categories (Supplementary Table 5). To examine the relative distribution of miRNA targeting across these pathways, we overlaid individual miRNA onto the enrichment map (Supplementary Figure 5). In this exploratory mapping, miR-182-5p was associated with genes across 66 nominally enriched GO terms, followed by miR-204-5p (45 terms), miR-2110 (44 terms), and miR-138-5p (42 terms). MiR-338-3p and miR-223-3p were linked to 27 and 26 GO terms, respectively, whereas miR-625-3p was associated with only four terms. Notably, miR-625-3p was detected in only a subset of samples (COPD *n* = 18, 75%; HV-ES *n* = 12, 60%), further limiting confidence in its inferred functional impact.

Seven out of eight miRNAs were predicted to target genes within at least one metabolic-related GO term, with miR-182-5p showing the broadest distribution across these categories. However, given that no enrichment results survived FDR correction and that the analysis is based on predicted and filtered interaction sets within a small cohort, these observations should be considered hypothesis-generating and intended to inform prioritization for future validation studies rather than to establish definitive pathway-level conclusions.

### Association of miRNA target gene expression with pathological changes in COPD

Given the prominence of metabolic processes among the most enriched GO terms, and the widespread targeting of genes within these pathways by dysregulated miRNAs, we next explored whether expression of miRNA-targeted metabolic genes were associated with structural lung abnormalities characteristic COPD. Building on prior work demonstrating significant correlations between differentially expressed lung EV miRNAs and key COPD disease features (16), we examined whether the predicted mRNA targets of these miRNAs exhibited similar relationships with quantitative CT measures of emphysema and SAD, assessed using both standard and novel imaging metrics (20).

**FIGURE 5:**
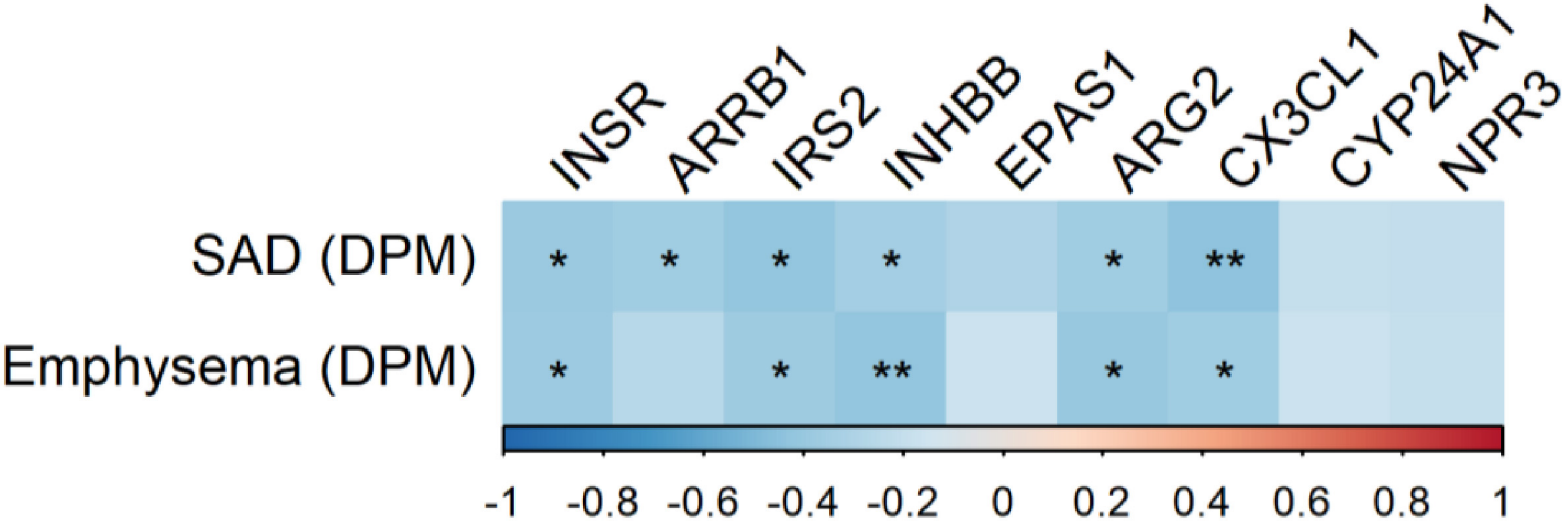
Correlation of metabolic-associated downregulated genes with CT-derived measures of airway disease. Correlation analysis was performed between downregulated genes involved in metabolic processes and airway measures obtained by CT, on those who had both measures (*N* = 42; COPD, *n* = 22; HV-ES, *n* = 20). Correlation analysis performed between log2 normalized miRNA and mRNA expression values using Spearman correlation coefficient using “*rcorr*” from *Hmisc* package (v5.1.1), statistical significance adjusted using BH to account for multiple testing using p.adjust from R “stats” (v4.3.2). Asterisk indicates statistical significance; *p < 0.05, **p < 0.01. BH, Benjamini–Hochberg; CT, computer tomography; DEG, differentially expressed genes; DPM, disease probability measures; SAD, small airways disease. Figure created using corrplot (v0.92) in R (v4.3.2).

Analyses were performed in the subset of subjects with paired CT imaging and epithelial RNA-sequencing data (*n* = 42), with COPD and HV-ES subjects pooled to capture the full, continuous spectrum of CT-derived lung abnormalities across the cohort. Within this pooled analytical framework, expression of five metabolic-associated genes (*INSR*, *IRS2*, *INHBB*, *ARG2*, and *CX3CL1*) showed significantly negative correlations with CT-derived measures of both SAD and emphysema (Figure 5), although associations may be influenced by the distinct CT phenotypes of the COPD and HV-ES subjects. Diagnosis-stratified scatter plots illustrating these associations are provided in the Supplementary Figure 6, demonstrating the distribution of relationships within each diagnostic group.

Consistent with previous findings, miR-182-5p was predicted to target the majority of these genes (*INSR*, *IRS2* and *CX3CL1*), while miR-223-3p and miR-200b-5p were predicted to target *INHBB* and *ARG2*, respectively. In addition, miR-2110 was predicted to target *INSR* alongside miR-182-5p, suggesting potential synergistic co-regulation of this gene by multiple miRNAs. Notably, these genes were also represented within other enriched GO terms mapping to biological clusters central to COPD pathogenesis, including immune cell regulation, cell signaling, actin organization, cell adhesion, and cell migration (Supplementary Table 5).

Collectively these findings highlight associations between reduced expression of miRNA-targeted metabolic genes and CT-defined structural lung abnormalities across the pooled study cohort. However, as COPD and HV-ES subjects differed in CT measures, these relationships may partly reflect between-group separation rather than independent continuous associations with disease severity. While exploratory in nature, and not adjusted for all potential confounding factors, these results support a potential link between miRNA-mediated metabolic dysregulation and epithelial dysfunction across the spectrum of COPD-related lung remodeling.

## Discussion

MicroRNAs are key modulators of cellular function and metabolic processes, and understanding their role in COPD could reveal novel biomarkers and therapeutic targets in the future. In this study, we expand on our previous findings of dysregulated lung-derived EV miRNAs from patients with COPD compared with HV-ES (16), to evaluate their potential regulatory effects on gene expression in matched epithelial brushings. Eight dysregulated miRNAs collectively mapped to 121 putative interactions with 191 DEGs in airway epithelium. Network visualization and enrichment analyses suggested enrichment of metabolic processes among miRNAs-targeted genes, with miR-182-5p consistently predicted to regulate multiple key DEGs. These findings provide novel, hypothesis-generating insights into EV miRNA-mediated regulatory networks in COPD.

Our findings build on our previous work (16) that differentially expressed lung-derived EV miRNAs, including miR-223-3p and miR-182-5p, may modulate disease relevant pathways. Although, miR-223-3p, was the most upregulated EV miRNA in COPD, miR-182-5p was predicted to target the largest number of DEGs, including several with greatest FCs in epithelial brushings such as *TLL1*, *ACTN2*, *NPR3*, and *COL4A4*. Some targets, notably *COL4A4* and *COL4A1*, were co-regulated by multiple miRNAs (miR-182-5p, miR-200b-5p and miR-2110), suggesting potential synergistic effects. Both genes encode subunits of type IV collagen, a major component of basement membranes and were downregulated in COPD epithelial brushings. *COL4A4* polymorphisms have been associated with reduced COPD risk in certain populations (41) and with regulation of the tumor microenvironment in lung adenocarcinoma (42). Given their role in basement membrane integrity and tissue remodeling, miRNA-mediated regulation of *COL4A4* and *COL4A1* may contribute to small vessel dysfunction, SAD, and emphysema in COPD.

Of the downregulated miRNA, we previously showed that miRNA-204-5p was the most significantly downregulated in the lung-derived EVs in COPD compared with HV-ES (log2FC −2.37, FDR 0.037) (16). In this study, miR-204-5p had the greatest number of putative mRNA targets (10.2% of the DEGs in epithelial brushings). Importantly, both miR-204-5p and miR-200b-5p (an upregulated EV miRNA) have been shown to regulate epithelial mesenchymal transition (EMT) and metastasis in tumor cells (43, 44). EMT has been implicated in the formation of peribronchial fibrosis in COPD and may be a precursor to lung cancer in these patients (45). Therefore, synergistic regulation of EMT by miR-204-5p and -200b-5p may contribute to the small airway fibrosis seen in COPD and predispose to early lung cancer.

Several of the upregulated genes, including *IL1R2, MMP12 and AKR1B10* have been previously implicated in COPD pathogenesis. IL1R2 is elevated in sputum from patients with COPD and may predict future exacerbations (46). The *IL1R2* gene encodes an IL-1 cytokine receptor that binds IL-1β, a potent activator of alveolar macrophages leading to MMP-9 production (47). *MMP12* plays a key role in emphysema and small airway remodeling (48) and its selective inhibition has been proposed as a potential therapeutic strategy (49), suggesting that miRNA-mediated post-transcriptional regulation could modulate its activity. *AKR1B10*, an aldo-keto reductase, regulates inflammatory cytokines such as IL-6 and IL-1β via stress-induced signaling pathways, including Nrf2 and MAPK, promoting inflammation (50). *AKR1B10* also contributes to lipid metabolism and fatty acid synthesis, which may further enhance inflammatory responses, and is activated by smoking in both COPD and healthy smokers (51), potentially linking it to lung cancer through autophagy dysregulation.

In addition, several downregulated genes in COPD epithelial brushings may also contribute to airway remodeling and systemic complications. *TLL1*, which encodes a metalloprotease involved in extracellular matrix degradation, showed reduced expression in our ex-smoker cohort. This contrasts with previous studies showing upregulation after acute smoke exposure, suggesting temporal or context-dependent regulation (52). CD34, essential for vascular endothelial repair, is also reduced in peripheral blood of current smokers (53) and patients with COPD (54). While *NPR3*, another downregulated gene in our dataset, has been linked to COPD pathogenesis (55), epigenetic alterations (56), and treatment responsiveness in preclinical models (57). *IGF-1*, similarly downregulated, is implicated in right heart failure (58) and in skeletal muscle wasting (59), raising the possibility that EV miRNAs may contribute to multimorbidity in COPD. Collectively, these findings highlight that dysregulated EV miRNAs may influence both local epithelial remodeling and systemic disease processes through coordinated regulation of key target genes.

Our analyses further explored whether expression of miRNA-targeted metabolic genes was associated with structural lung abnormalities characteristic of COPD. Among subjects with paired epithelial RNA-sequencing and CT imaging (*n* = 42), expression of five metabolism-associated genes – *INSR, IRS2, INHBB, ARG2*, and *CX3CL1* – showed inverse correlations with CT-derived measures of SAD and emphysema across the pooled cohort. However, as these analyses were performed across pooled groups with distinct CT phenotypes, the observed relationships should not be interpreted as definitive evidence of continuous severity-associated effects within COPD alone. Previous murine airway models have reported that modulation of INSR alleviates airway hyperviscosity, inflammatory infiltration, and airway remodeling (60), and IRS2 modulation enhances pulmonary inflammation, accumulation of alveolar macrophages, and airway and vascular remodeling upon allergen stimulation (61); although these findings derive from experimental systems and may not directly translate to human COPD biology.

With respect to CX3CL1, differences in reported expression across studies may reflect context-dependent regulation, including differences in sample type, disease stage, and cellular compartment. In our dataset, CX3CL1 was reduced in bronchial epithelium compared with HV-ES, whereas prior studies have reported increased circulating levels in COPD and associations with markers of systemic inflammation and small airway obstruction COPD (62). Furthermore, exposure to cigarette smoke was shown to upregulate *CX3CL1* gene expression mainly in macrophage and T lymphocyte cells potentially promoting CX3CR1^+^ cell accumulation within the lungs during both acute and chronic inflammatory stress (63). Together, these findings suggest that CX3CL1 regulation may be cell-type specific in COPD, although this remains speculative.

miR-182-5p was predicted to target several of these genes, with miR-223-3p, miR-200b-5p and miR-2110 potentially contributing to broader regulatory interactions within the network. While pathway and GO analyses suggested possible enrichment in processes including immune regulation, cell signaling, cytoskeletal organization, and cell adhesion, these did not remain significant after correction for multiple testing and are therefore interpreted as exploratory only.

A major strength of this work is the paired dataset between the EV miRNA and epithelial brushings from the same lung lobe, in the same subject, focusing on the airway epithelium’s central role as an immune-sensing and inflammatory orchestrator in COPD pathogenesis (4) to enable biologically meaningful, spatially matched comparisons within the same local microenvironment. This enables a more accurate description of the miRNA-mRNA interactions, rather than relying on *in silico* analysis alone or even experimental models, which may oversimplify disease processes (64). The combinatorial analytical approach - incorporating correlation, synergistic analysis, network construction, and visualization - emphasized biologically relevant interactions and highlighted targets suitable for further validation. Our study consisted of well-characterized patients with mild-to-moderate COPD, providing insights into early-stage disease. Replication studies and analyses in more severe COPD are now warranted to further delineate the role of miRNAs in disease progression.

Our study has several limitations. The sample size was modest, particularly for GO enrichment and CT association analyses, and some miRNAs, such as miR-625-3p, were only detected in a subset of samples, limiting interpretability. In addition, pathway and GO enrichment analyses did not retain statistical significance after correction for multiple testing and should therefore be interpreted as exploratory and hypothesis-generating rather than confirmatory. Many associations are correlation-based and should not be interpreted as causal. CT-derived disease probability measures and gene expression were analyzed across the pooled COPD and HV-ES cohort. Given the marked differences in CT abnormalities between groups, some observed associations may reflect diagnostic group separation rather than continuous disease-severity relationships within COPD. Network construction assumptions, thresholding choices, and reliance on predicted or partially validated miRNA-mRNA interactions should be considered when interpreting results. The designation of miR-182-5p as a potential regulator refers to its putative role within this specific DEG-constrained network that contains only miRNA-mRNA interactions. As such, this analysis does not capture interactions between mRNAs, between miRNAs, or additional regulatory layers such as transcription factors, protein-protein interactions, or post-transcriptional modifications. Moreover, the network is constrained to a pre-selected set of differentially expressed genes and miRNAs, so additional miRNAs or mRNAs not included in the dataset could influence overall network topology. Therefore, while miR-182-5p emerges as the most highly connected node in this network its inferred regulatory importance should be interpreted within the context of the specific experimental and analytical framework used. Importantly, as the findings presented here are based on computational integration and correlation analyses and do not establish causal or functional miRNA–mRNA regulatory relationships. Experimental validation will be required to determine the biological significance of these interactions in COPD.

In addition, EV characterization in this study, while performed using complementary approaches (including CD9 ELISA, transmission electron microscopy, and assessment of EV-associated and non-EV protein markers), remains limited relative to current reporting standards. In particular, particle size distribution and concentration measurements (e.g., nanoparticle tracking analysis) were not performed due to sample size and instrumentation constraints. As a result, we were unable to normalize RNA content to EV particle number. Although particle-based normalization is increasingly reported, there is currently no consensus on optimal normalization strategies in EV research, and such approaches may obscure biologically relevant differences in EV yield between disease states. Furthermore, as EV particle concentration was not quantified, it remains uncertain whether the observed differences in EV-associated miRNA expression reflect altered miRNA cargo per vesicle, differences in total EV abundance, or both. Consequently, interpretation of the EV-miRNA findings should be made cautiously. To minimize technical variability, we standardized the starting BALF volume across all samples; however, the above factors should be considered when interpreting the EV-derived RNA findings.

Cumulative smoking exposure was not included as a covariate in the differential expression analysis so may represent a limitation in the interpretation of the outcomes in this work. While the restriction to ex-smokers reduces the impact of acute smoking-related effects, some contribution of prior smoking exposure to the observed transcriptional differences cannot be entirely ruled out. The COPD patients in this study had relatively mild disease (mean FEV1% predicted 77.5 ± 14.8), unlike previous EV miRNA studies (65–67), which included more severe cases and current smokers. While those findings may apply to a broader COPD population, their mechanistic relevance is limited by patient heterogeneity and confounding from active smoking. Moreover, these prior studies used blood or sputum samples, which do not reliably reflect lung tissue changes (68). In contrast, our use of BAL-derived EV miRNA offers a more direct link to lung-specific transcriptomic alterations and provides biologically relevant insight into EV-associated regulatory mechanisms in COPD.

In conclusion, our work highlights candidate pathways potentially regulated by lung-derived EV miRNAs, particularly pathways linked to metabolic processes, structural integrity, and epithelial remodeling. These findings generate testable hypotheses for experimental validation, including functional assays to confirm miRNA-target interactions and mechanistic studies in cell and animal models. Ultimately, understanding EV miRNA-mediated regulation may provide insights into early disease mechanisms, and identify candidate pathways and biomarkers for future mechanistic and translational investigation in COPD.

## Data Availability

The datasets presented in this study can be found in online repositories. The names of the repository/repositories and accession number(s) can be found in the article/Supplementary material.
