# Supplementary material for: Integrated miRNA–mRNA network analysis identifies miR-182-5p as a potential regulator in COPD pathogenesis
Source: Front Med (Lausanne). 2026 Jun 22;13:1743277. doi: 10.3389/fmed.2026.1743277 (PMC13333699; doi:10.3389/fmed.2026.1743277)
Supplement: Supplementary file 1 [file Data_Sheet_1.pdf]

Appendix 1:

*MICAII study group

Bastian Angerman [1]
Stephanie Ashenden [2]
Sarah Bawden [3]
Graham Belfield [2]
Maria G. Belvisi [1,4]
Aurelie Bornot [2]
Jerome Bouquet [5]
Hannah Burke [3, 6]
Carolina Caceres [5]
Raghothama Chaerkady [7]
Doriana Cellura [3, 6]
Chia-Chien Chiang [8]
Kerry Day [3, 6]
Antonio DiGiandomenico [5]
Hanna Duàn [1]
Ulrika Edvardsson [9]
Damla Etal [2]
Anna Freeman [3, 6]
Matthew S. Glover [7]
Vancheswaran Gopalakrishnan [5]
Stephen Harden [10]
Sonja Hess [7]
Alex Hicks [3, 6]
Ventzislava A. Hristova [7]
Michael Hühn [1]
Fredrik Karlsson [2]
Shameer Khader [8]
Glenda Lassi [1]
Alex Mackay [1,4]
Christopher McCrae [1]
Christopher Morehouse [5]
Daniel Muthas [1]
Karl Nordström [2]
Steven Novick [2]
Esther Nyimbili [3]
Kristoffer Ostridge [1, 6]
Lisa Öberg [1]
Adam Platt [14]
Laura Presland [3]
Xiaotao Qu [8]
Nicola Rayner [3]
Pedro Rodrigues [3]
Bret Sellman [5]
Gary Sims [1]
Cosma Mirella Spalluto [6]
Andria Staniford [3]
Karl J. Staples [3, 6]
Bruce Thompson [12]
Outi Vaarala[13]
Junmin Wang [7]
Paul Warrener [5]
Alastair Watson [6]
Nicholas P. Williams [3, 6]
Tom M. A Wilkinson [3, 6]
Wen Yu [8]
Bairu Zhang [2]
Tianhui Zhang [2]
Natalie van Zuydam [2]

[1] Research and Early Development, Respiratory & Immunology, BioPharmaceuticals R&D, AstraZeneca, Gothenburg, Sweden
[2] Translational Genomics, Discovery Biology, Discovery Sciences, BioPharmaceuticals R&D, AstraZeneca, Gothenburg, Sweden
[3] NIHR Southampton Biomedical Research Centre, Southampton, UK
[4] National Heart & Lung Institute, Imperial College London, London, UK
[5] Microbial Sciences, BioPharmaceuticals R&D, AstraZeneca, Gothenburg, Sweden
[6] Faculty of Medicine, University of Southampton, Southampton, UK
[7] Dynamic Omics, Centre for Genomics Research, Discovery Sciences, BioPharmaceuticals R&D, AstraZeneca, Gaithersburg, USA
[8] Data Science and Artificial Intelligence, BioPharmaceuticals R&D, AstraZeneca, Gothenburg, Sweden
[9] Business Development and Licensing, BioPharmaceuticals R&D, AstraZeneca, Gothenburg, Sweden
[10] University Hospital Southampton NHS Foundation Trust, Southampton, UK
[11] Research and Early Development, Respiratory & Immunology, BioPharmaceuticals R&D, AstraZeneca, Gaithersburg, USA
[12] Swinburne University of Technology Melbourne, Australia
[13] Faculty of Medicine, University of Helsinki, Helsinki, Finland
[14] Research and Early Development, Respiratory & Immunology, BioPharmaceuticals R&D, AstraZeneca, Gaithersburg, USA

Supplementary Methods

Bronchoalveolar lavage fluid analysis

Following separation of the cell-free supernatant (described in the main Methods), the remaining BALF cell pellet was processed for cytological analysis. The pellet was resuspended in 10 mL hypotonic lysis buffer for 2 minutes to remove red blood cell contamination. Subsequently, 10 mL of hypertonic recovery buffer was added, and the volume was adjusted to 40 mL with 1× phosphate-buffered saline (PBS). The sample was centrifuged at 400 g for 10 minutes.

The resulting pellet was resuspended in 1 mL 1× PBS, and total cell counts were determined using the Trypan blue exclusion method. The cell suspension was then adjusted to a concentration of 0.5 × 10^6 cells/mL. A volume of 75 µL was loaded into cytospin funnels and centrifuged at 350 g for 6 minutes onto Poly-L-lysine-coated slides.

Slides were air-dried overnight and stained the following day using the Rapid Romanowsky A–B–C kit (TCS Biosciences, Buckingham, UK). Differential cell counts were performed by enumerating 500 cells per slide under light microscopy at ×40 magnification. Absolute counts and relative percentages of eosinophils, neutrophils, macrophages, lymphocytes, bronchial epithelial cells, and squamous cells were calculated.

EV RNA isolation, library preparation and small RNA sequencing of BAL EVs performed by QIAGEN® Genomic Services

To further assess RNA extraction and downstream assay performance, Qiaseq miRNA Library QC spike-ins (52 synthetic RNAs) were added to each lysed EV sample prior to extraction.

Reverse transcription was performed in 10 µL reactions using the miRCURY LNA RT kit (Qiagen®), incorporating the UniSp6 artificial RNA spike-in to monitor reverse transcription efficiency. Following cDNA synthesis, quantitative PCR was carried out on a LightCycler® 480 Real-Time PCR System (Roche®, Welwyn Garden City, UK) using 384-well plates. Assays included endogenous miRNAs (miR-23a, miR-30c, miR-103, miR-142-3p, miR-451), the 52 spike-in controls (Table 2.3), and UniSp6. No-template controls were included and processed identically to samples. Amplification curves were analysed using LightCycler® software, with Cq values determined by the second derivative method and specificity confirmed by melting curve analysis.

For library preparation, due to low RNA input (1 ng), the Qiaseq miRNA NGS 3' adapter was diluted 1:10 in nuclease-free water prior to use. Library quality and size distribution were assessed using a high-sensitivity DNA chip on an Agilent® Bioanalyzer 2100, and concentrations were measured using a Qubit™ Fluorimeter (Thermo Fisher Scientific). Individual libraries were normalised to 4 nM, pooled in equimolar amounts, and denatured prior to sequencing. Sequencing was performed at a final loading concentration of 1.8 pM, generating an average of 2.8 million reads per sample.

Trimming of adaptors and UMI correction performed by Qiagen® Genomic Services

Sequencing data was demultiplexed, and BCL files converted to standard FASTQ file format for downstream analysis using bcl2fastq conversion software v2.20 (Illumina®). Next, cutadapt (v1.11) is used to remove low quality bases and identify the adapter and UMIs applied during library. The output from cutadapt is used to remove adapter sequences and to collapse reads by unique molecular index (UMI) with in-house script. Each raw read is expected

to contain (starting from the 5' end): an insert sequence, the adapter sequence, 12nt-long UMI sequence, and other ligated sequence. Only reads that contain adapters, have insert sequences ≥16 nucleotides and a UMI length ≥10 nucleotides are kept. Insert sequences from reads which do not contain full length UMI sequence are output as "partial-UMI reads". Full IMU length reads with identical insert and UMI sequences are merged and then combined with partial-UMI reads as output of UMI correction (summarized in flowchart below).

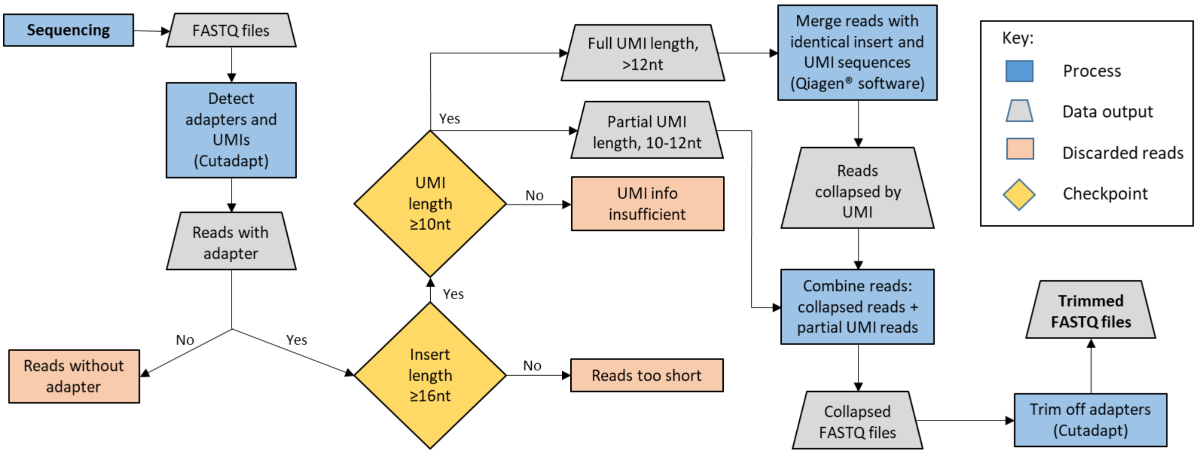

miRNA sequencing quality control, mapping and alignment

Trimmed FASTQ files were analysed for read quality using FastQC tool. Bowtie2 (v2.2.2) tool was used to align sequencing reads to the reference genome (GRCh37/hg19), and miRNA to the miRNA database, miRBase (version mirbase_20).

miRNA differential expression analysis

Differential gene expression were assessed with edgeR (v3.14.0) in R (v3.6.2). Prior to analysis, lowly expressed miRNAs were filtered from the dataset using a threshold of CPM <10 in at least 15 samples. miRNA expression data were filtered to improve reliability and biological relevance prior to downstream analysis. miRNAs with low expression across samples were excluded, as these provide limited statistical power for detecting differential expression and may violate assumptions of downstream methods due to high discreteness. Filtering was performed using counts per million (CPM) to account for differences in library size between samples. Three filtering approaches were evaluated: (i) a median $\log_2$CPM threshold, (ii) CPM >1 in at least 15 samples, and (iii) CPM >10 in at least 15 samples. The performance of each method was assessed using graphical analyses, including boxplots and principal component analysis (PCA), to evaluate data distribution and identify outliers. The CPM >10 in at least 15 samples threshold was selected, as it reduced low-expression noise and enabled more consistent identification of outliers, resulting in a dataset suitable for downstream differential expression analysis.

Next, reads underwent normalisation using the calcNormFactorsfunction in edgeR which normalises for RNA composition by finding a set of scaling/normalisation factors for the library sizes that minimise the log-fold changes between the samples for most miRNA. The default method for computing these scale factors uses a trimmed mean of M-values (TMM) between each pair of samples. The normalisation factors of all the libraries multiply to unity. The TMM-normalised dataset was then used for the differential expression analysis between patients with COPD and healthy volunteer ex-smokers (HV-ES).

The following code performs the TMM normalisation and generates a list of normalisation factors for each sample, where “keep” is the log-transformed CPM dataset with the lowly expressed miRNA removed.

```
> TMM_normalised <- calcNormFactors(keep)
> TMM_normalised$samples
```

Negative binomial distribution methods were used to model the TMM normalized dataset. The following code was used to calculate dispersion estimates where “TMM_normalised” is the TMM normalised miRNA dataset and “design” is a model matrix based on the experimental design of the study (i.e. samples either assigned to COPD or Healthy).

```
> Condition <- factor(group[ ,”Disease”], levels=c(“Healthy”, “COPD”)
> design <- model.matrix(~Condition)
> y <- estimateDisp(TMM_normalised,design)
## The square root of the common dispersion gives the BCV
> sqrt(y$common.dispersion)
```

The common dispersion estimate was calculated as 0.398 (~0.4 is usual for biological studies). Trended dispersion estimates and miRNA specific estimates (referred to as “Tagwise” in edgeR) were used in testing for differential expression. Once negative binomial models were fitted and dispersion estimates obtained, edgeR determined differential miRNA expression using the exact test. The following code was used to determine differential miRNA expression, where “y” is the dispersion estimates calculated above:

```
>  et <- exactTest(y)
## To give the top 10 differentially expressed miRNA
> topTags(et)
## To list the miRNA differentially expressed at a false discovery rate (FDR) of 5%
> results_edgeR <- topTags (et, n= nrow(data_clean), sort.by = “none”)
> sum(results_edgeR$table$FDR<0.05)
## To visualise the data on an MA plot, showing the log2 fold change on y axis
versus average log 2 CPM on x axis for differentially expressed miRNA, with miRNA
with an FDR<0.05 in red.
> plotSmear (et, de.tags  = rownames (results_edgeR)
[results_edgeR$table $FDR<0.05], pch=16, cex=1)
## Additional information can be added to the MA plot e.g. blue
line representing two-fold change in expression
> abline(h=c(-1,1), col = “blue”)
```

RT-qPCR confirmation of RNA sequencing results

To confirm the findings of the BAL EV RNA sequencing results, RT-qPCR was performed on 44 BAL EV samples for a total of 46 assays, including 6 stably expressed miRNA for normalization and 5 RNA spike-ins to assess RNA isolation efficiency and the quality of the reverse transcription reaction.

NormFinder software in Microsoft® Office Excel was used to identify miRNA that were most stably expressed across all samples from the miRNA sequencing results. These miRNAs were then used as normalisers for the qPCR validation study. Although all of these “normaliser” miRNA were measured by RT-qPCR, only “normaliser” miRNA detected in all samples were used for normalisation of Cq data.

Total RNA was isolated from BAL EVs as stated previously. Reverse transcription was performed in 10μL reactions using the miRCURY LNA RT kit (Qiagen®). Following cDNA synthesis, qPCR was performed in a LightCycler® 480 Real-Time PCR System (Roche®) in 384 well plates. Amplification curves were analysed using the Roche LC software, for both determination of Cq (by the 2nd derivative method) and for melting curve analysis. The mean Cq for all the universally expressed "normaliser" miRNA was calculated to give a Geomean Cq. Then, the following formula was used to calculate the normalized Cq values:
Normalized Cq of miRNA of interest (ΔCq) = Geomean Cq – miRNA of interest Cq
A higher value thus indicates that the miRNA is more abundant in the particular sample. Values were then presented as 2ΔCq to represent fold change.

The EV miRNA sequencing data access - The data presented in this study are deposited in the GEO repository, accession number: GSE218571.

RNA sequencing of epithelial brushings performed by AstraZeneca

Library preparation was performed across four separate runs (one 96-well plate per run), generating four independent library pools. Following pooling, libraries were quantified using a Qubit Fluorimeter (Thermo Fisher Scientific) and fragment size distribution was confirmed using a Fragment Analyzer (Agilent Technologies).

Three of the four library pools were diluted to 1 nM and sequenced on a NovaSeq 6000 (Illumina®) using the NovaSeq 6000 S4 reagent kit (2 × 76 cycles). The remaining pool was diluted to 1.9 nM and sequenced on a NovaSeq 6000 using two NovaSeq 6000 SP S1 reagent kits (2 × 51 cycles).

Across all samples, sequencing generated an average of 52.6 million reads per sample.

RNA sequencing data access: The full clinical dataset and RNA sequencing dataset generated from analysis of the epithelial brushings, are not publicly available to protect the privacy of all individuals whose data we have collected, stored, and analysed. However, data may be made available upon reasonable request by applying through the established Data Request Portal through which Researchers can request access to de-identified clinical data (https://vivli.org), after which, clinical data may be made available upon review of the patient consent forms, scientific merit of the proposal, and signature of a data sharing/collaboration agreement. This mechanism allows controlled, risk managed accessibility of the data and at the same time safeguards subjects' confidentiality.

Integrative analysis of miRNA–mRNA interactions

An overview of the methods used to identify putative miRNA-mRNA interactions and the subsequent network and functional enrichment analysis. The Blue rectangles indicate a process with the software/tool specified in parenthesis, the grey trapeziums indicate a data output, and the yellow diamond an important checkpoint.

Workflow for miRNA-mRNA network construction and analysis:

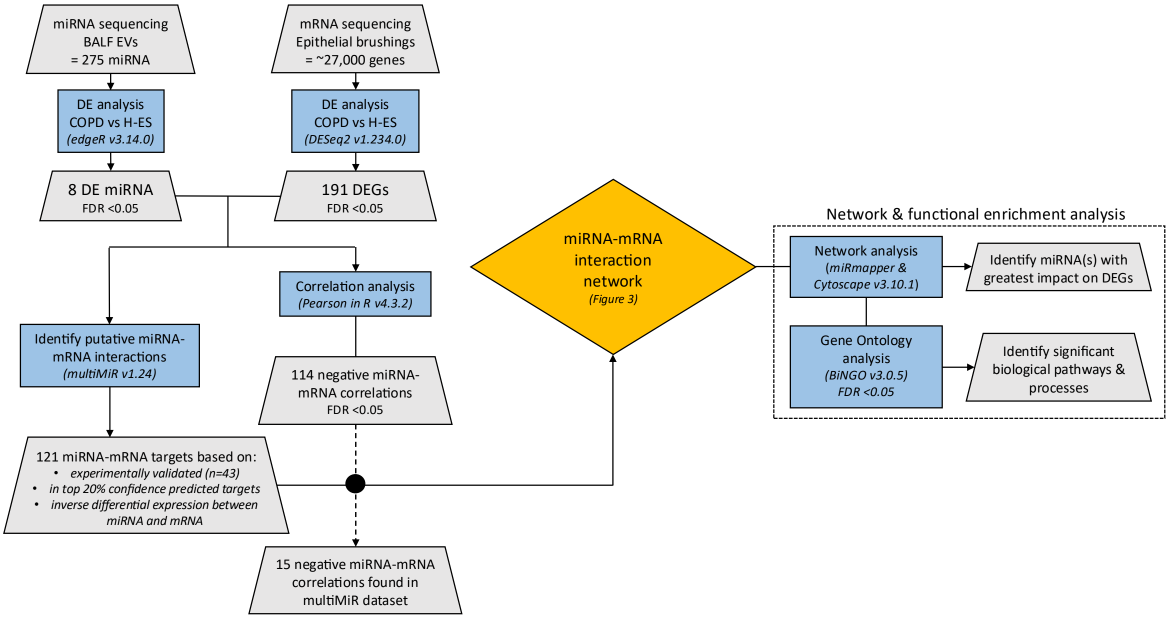

Supplementary data

Table S1. Significantly differentially expressed miRNA measured by RT-qPCR between COPD subjects and HV-ES, N=44

miRNA	COPD SD	HV_ES SD	Log2FC	P value	FDR
**Up-regulated in COPD**					
**hsa-miR-2110**	1.21	0.71	2.12	0.001	0.016
**hsa-miR-223-3p**	1.47	1.37	2.97	0.001	0.016
**hsa-miR-625-3p†**	0.91	0.76	1.85	0.006	0.041
**hsa-miR-182-5p**	0.70	0.66	1.52	0.006	0.041
**hsa-miR-200b-5p**	0.79	0.72	1.52	0.009	0.047
**Down-regulated in COPD**					
**hsa-miR-204-5p**	1.32	1.23	-2.37	0.003	0.037
**hsa-miR-138-5p**	0.90	0.77	-1.66	0.005	0.041
**hsa-miR-338-3p**	1.15	0.78	-1.72	0.009	0.047

Shapiro-Wilk test for normality was performed and showed data were normally distributed. Unpaired Welch's t test was performed and then adjusted using Benjamini Hochberg to generate an FDR value.
†missing data points; COPD, n=18; HV-ES, n=12
COPD, Chronic obstructive pulmonary disease, FC: Fold change. FDR, false discovery rate; HV-ES, healthy volunteer ex-smoker; miRNA, microRNA, SD: standard deviation.

Table S2. Differentially expressed genes in epithelial brushings comparing COPD subjects with HV-ES

ENSEMBL ID	HGNC SYMBOL	Log2FC	P-value	FDR
ENSG00000262526		27.47	1.12E-16	3.23E-12
ENSG00000287059		2.14	5.81E-08	0.000261
ENSG00000238266	*LINC00707*	2.12	5.65E-07	0.001354
ENSG00000115590	*IL1R2*	2.01	2.00E-07	0.000575
ENSG00000198074	*AKR1B10*	1.74	2.27E-08	0.000163
ENSG00000180438	*TPRXL*	1.72	6.00E-10	8.63E-06
ENSG00000198488	*B3GNT6*	1.6	6.58E-06	0.006767
ENSG00000287771		1.6	1.04E-05	0.008777
ENSG00000111700	*SLCO1B3*	1.6	7.40E-06	0.007098
ENSG00000105388	*CEACAM5*	1.55	1.38E-06	0.002094
ENSG00000145934	*TENM2*	1.54	3.57E-06	0.004526
ENSG00000100055	*CYTH4*	1.48	9.28E-07	0.001487
ENSG00000231683		1.47	1.10E-05	0.008777
ENSG00000182885	*ADGRG3*	1.44	1.67E-05	0.010448
ENSG00000253339		1.43	7.99E-08	0.000261
ENSG00000134827	*TCN1*	1.42	1.37E-05	0.009656
ENSG00000255833	*TIFAB*	1.31	3.00E-05	0.014412
ENSG00000164303	*ENPP6*	1.29	3.23E-05	0.014755
ENSG00000163421	*PROK2*	1.28	3.21E-05	0.014755
ENSG00000151012	*SLC7A11*	1.26	1.07E-05	0.008777
ENSG00000262406	*MMP12*	1.25	2.30E-05	0.012952
ENSG00000176697	*BDNF*	1.22	2.60E-05	0.013383
ENSG00000118785	*SPP1*	1.21	4.59E-05	0.018715
ENSG00000289013		1.2	4.67E-05	0.018715
ENSG00000146013	*GFRA3*	1.2	1.90E-05	0.011391
ENSG00000080031	*PTPRH*	1.2	2.01E-05	0.01178
ENSG00000065618	*COL17A1*	1.19	8.43E-07	0.001487
ENSG00000137440	*FGFBP1*	1.17	5.07E-05	0.019647
ENSG00000160862	*AZGP1*	1.16	1.26E-05	0.009519
ENSG00000103888	*CEMIP*	1.16	2.16E-05	0.012425
ENSG00000273331	*TM4SF19-DYNLT2B*	1.16	4.57E-05	0.018715
ENSG00000270164	*LINC01480*	1.14	5.80E-05	0.021298
ENSG00000102962	*CCL22*	1.13	6.38E-05	0.022137
ENSG00000206069	*LHFPL7*	1.12	2.48E-05	0.013185
ENSG00000106258	*CYP3A5*	1.09	2.75E-06	0.003767
ENSG00000274008	*Metazoa_SRP*	1.08	7.07E-05	0.023647
ENSG00000176153	*GPX2*	1.07	7.02E-07	0.001487
ENSG00000232079	*LINC01697*	1.06	1.06E-05	0.008777
ENSG00000104783	*KCNN4*	1.05	5.08E-06	0.006094
ENSG00000134757	*DSG3*	0.99	3.66E-05	0.016443
ENSG00000090659	*CD209*	0.95	5.19E-05	0.019647
ENSG00000146592	*CREB5*	0.94	7.92E-05	0.025219
ENSG00000272405		0.94	0.000128	0.034507
ENSG00000248323	*LUCAT1*	0.94	8.02E-07	0.001487
ENSG00000283994		0.92	0.000102	0.029947
ENSG00000158481	*CD1C*	0.9	0.000156	0.038086
ENSG00000232931	*LINC00342*	0.89	1.82E-05	0.011169
ENSG00000228971	*LINC02607*	0.89	0.000127	0.034507
ENSG00000162433	*AK4*	0.87	2.51E-05	0.013185
ENSG00000203685	*STUM*	0.87	0.000175	0.038945
ENSG00000166897	*ELFN2*	0.86	0.000165	0.038945
ENSG00000127507	*ADGRE2*	0.85	0.000175	0.038945

ENSEMBL ID	HGNC SYMBOL	Log2FC	P-value	FDR
ENSG00000260997		0.85	0.000172	0.038945
ENSG00000241186	*TDGF1*	0.84	0.000195	0.040696
ENSG00000148926	*ADM*	0.81	0.0002	0.041032
ENSG00000196344	*ADH7*	0.79	5.93E-05	0.021342
ENSG00000151632	*AKR1C2*	0.78	5.17E-05	0.019647
ENSG00000234292		0.78	0.000242	0.043194
ENSG00000251194		0.78	0.000184	0.039749
ENSG00000121769	*FABP3*	0.78	0.000234	0.042203
ENSG00000180861	*LINC01559*	0.78	0.000244	0.043347
ENSG00000144452	*ABCA12*	0.77	0.000207	0.041032
ENSG00000253123		0.77	0.000233	0.042203
ENSG00000249359		0.76	0.000211	0.041032
ENSG00000010327	*STAB1*	0.76	0.00021	0.041032
ENSG00000155918	*RAET1L*	0.75	0.000127	0.034507
ENSG00000105641	*SLC5A5*	0.75	0.000271	0.045549
ENSG00000237523	*LINC00857*	0.74	0.000197	0.040696
ENSG00000171004	*HS6ST2*	0.73	3.90E-05	0.017014
ENSG00000288853		0.71	0.000152	0.037651
ENSG00000152256	*PDK1*	0.71	5.99E-06	0.006626
ENSG00000246203		0.69	0.000276	0.045549
ENSG00000146267	*FAXC*	0.69	9.57E-05	0.028979
ENSG00000137462	*TLR2*	0.69	0.000221	0.041491
ENSG00000139364	*TMEM132B*	0.69	0.000267	0.045311
ENSG00000179593	*ALOX15B*	0.68	0.00025	0.04389
ENSG00000158292	*GPR153*	0.68	0.000133	0.034791
ENSG00000026508	*CD44*	0.67	9.95E-05	0.029529
ENSG00000154277	*UCHL1*	0.66	0.000219	0.041491
ENSG00000258227	*CLEC5A*	0.65	0.000273	0.045549
ENSG00000280434		0.65	0.000279	0.045549
ENSG00000232977	*LINC00327*	0.64	0.000321	0.048907
ENSG00000251209	*LINC00923*	0.63	0.000114	0.032872
ENSG00000136542	*GALNT5*	0.6	0.00012	0.033983
ENSG00000029153	*BMAL2*	0.58	0.000144	0.037071
ENSG00000184731	*FAM110C*	0.56	0.000169	0.038945
ENSG00000167553	*TUBA1C*	0.55	0.00019	0.040413
ENSG00000121380	*BCL2L14*	0.54	6.85E-05	0.023195
ENSG00000118242	*MREG*	0.51	0.000193	0.040696
ENSG00000149043	*SYT8*	0.51	0.000318	0.048907
ENSG00000235151		0.49	0.000194	0.040696
ENSG00000134285	*FKBP11*	0.48	0.000147	0.037071
ENSG00000144045	*DQX1*	0.46	0.00031	0.048214
ENSG00000112699	*GMDS*	0.46	7.64E-05	0.024704
ENSG00000075240	*GRAMD4*	0.46	0.000171	0.038945
ENSG00000166401	*SERPINB8*	0.45	0.000304	0.048214
ENSG00000101311	*FERMT1*	0.42	0.000148	0.037071
ENSG00000135090	*TAOK3*	0.41	0.000223	0.041491
ENSG00000112972	*HMGCS1*	0.39	0.000173	0.038945
ENSG00000115295	*CLIP4*	0.38	3.00E-05	0.014412
ENSG00000158715	*SLC45A3*	0.38	0.00022	0.041491
ENSG00000259642	*ST20-AS1*	0.37	0.000309	0.048214
ENSG00000095261	*PSMD5*	0.35	0.000224	0.041491
ENSG00000151835	*SACS*	0.34	0.000325	0.049166
ENSG00000171492	*LRRC8D*	0.23	0.000329	0.04956
ENSG00000149311	*ATM*	0.22	0.000297	0.047679
ENSG00000083223	*TUT7*	0.22	0.000277	0.045549
ENSG00000077684	*JADE1*	-0.19	0.000223	0.041491

ENSEMBL ID	HGNC SYMBOL	Log2FC	P-value	FDR
ENSG00000136068	*FLNB*	-0.2	0.000173	0.038945
ENSG00000176842	*IRX5*	-0.2	0.00028	0.045549
ENSG00000109762	*SNX25*	-0.27	0.000315	0.048689
ENSG00000047648	*ARHGAP6*	-0.33	0.000307	0.048214
ENSG00000112773	*TENT5A*	-0.33	0.00013	0.034572
ENSG00000171105	*INSR*	-0.34	6.08E-05	0.02152
ENSG00000118960	*HS1BP3*	-0.36	6.41E-08	0.000261
ENSG00000143365	*RORC*	-0.39	9.30E-07	0.001487
ENSG00000214357	*NEURL1B*	-0.4	3.11E-05	0.01467
ENSG00000005882	*PDK2*	-0.4	2.70E-05	0.013418
ENSG00000141232	*TOB1*	-0.4	0.000181	0.039558
ENSG00000137486	*ARRB1*	-0.41	7.63E-05	0.024704
ENSG00000185950	*IRS2*	-0.45	0.000308	0.048214
ENSG00000158352	*SHROOM4*	-0.45	0.00025	0.04389
ENSG00000132326	*PER2*	-0.47	5.12E-05	0.019647
ENSG00000181690	*PLAG1*	-0.47	0.000133	0.034791
ENSG00000117479	*SLC19A2*	-0.47	9.49E-06	0.008777
ENSG00000116016	*EPAS1*	-0.48	0.00021	0.041032
ENSG00000163083	*INHBB*	-0.48	8.37E-05	0.026192
ENSG00000144668	*ITGA9*	-0.48	0.000124	0.034257
ENSG00000152377	*SPOCK1*	-0.48	0.000267	0.045311
ENSG00000184986	*TMEM121*	-0.48	0.000159	0.038529
ENSG00000159409	*CELF3*	-0.49	0.000264	0.045311
ENSG00000178904	*DPY19L3*	-0.49	5.51E-06	0.006348
ENSG00000244921	*MTCYBP18*	-0.49	0.000298	0.047679
ENSG00000119698	*PPP4R4*	-0.49	0.000135	0.035065
ENSG00000155090	*KLF10*	-0.5	1.35E-05	0.009656
ENSG00000112759	*SLC29A1*	-0.5	0.000255	0.044212
ENSG00000181458	*TMEM45A*	-0.5	0.000208	0.041032
ENSG00000169515	*CCDC8*	-0.51	8.58E-05	0.026537
ENSG00000265933	*LINC00668*	-0.51	0.000225	0.041491
ENSG00000229337		-0.52	0.000207	0.041032
ENSG00000173706	*HEG1*	-0.54	0.000268	0.045311
ENSG00000138615	*CILP*	-0.55	0.000214	0.041285
ENSG00000177283	*FZD8*	-0.56	1.20E-05	0.009343
ENSG00000205835	*GMNC*	-0.57	0.000321	0.048907
ENSG00000146197	*SCUBE3*	-0.57	7.97E-05	0.025219
ENSG00000116039	*ATP6V1B1*	-0.58	2.52E-05	0.013185
ENSG00000081181	*ARG2*	-0.59	0.000208	0.041032
ENSG00000006210	*CX3CL1*	-0.59	4.24E-05	0.018219
ENSG00000126562	*WNK4*	-0.62	0.000295	0.047679
ENSG00000288930		-0.65	6.67E-05	0.022861
ENSG00000171943	*SRGAP2C*	-0.67	5.85E-05	0.021298
ENSG00000263063		-0.7	0.000255	0.044212
ENSG00000152931	*PART1*	-0.7	3.82E-05	0.016895
ENSG00000255471	*PRSS23-AS1*	-0.71	0.000148	0.037071
ENSG00000137726	*FXYD6*	-0.73	0.00028	0.045549
ENSG00000198300	*PEG3*	-0.73	0.000122	0.03415
ENSG00000214870	*LINC02981*	-0.74	0.000233	0.042203
ENSG00000154864	*PIEZO2*	-0.74	0.000156	0.038086
ENSG00000130988	*RGN*	-0.74	0.000164	0.038945
ENSG00000166828	*SCNN1G*	-0.74	1.48E-05	0.009656
ENSG00000158050	*DUSP2*	-0.77	0.00021	0.041032
ENSG00000231754		-0.78	0.000235	0.042203
ENSG00000157570	*TSPAN18*	-0.8	0.000188	0.040386
ENSG00000110900	*TSPAN11*	-0.81	4.68E-05	0.018715

ENSEMBL ID	HGNC SYMBOL	Log2FC	P-value	FDR
ENSG00000019186	*CYP24A1*	-0.82	9.32E-05	0.028536
ENSG00000161055	*SCGB3A1*	-0.82	7.54E-05	0.024704
ENSG00000197838	*CYP2A13*	-0.83	2.42E-05	0.013185
ENSG00000168874	*ATOH8*	-0.84	1.48E-05	0.009656
ENSG00000187498	*COL4A1*	-0.84	0.000176	0.038945
ENSG00000125144	*MT1G*	-0.85	9.92E-05	0.029529
ENSG00000168917	*SLC35G2*	-0.85	0.000168	0.038945
ENSG00000197291	*RAMP2-AS1*	-0.86	6.56E-06	0.006767
ENSG00000129437	*KLK14*	-0.87	0.000105	0.03045
ENSG00000162426	*SLC45A1*	-0.88	0.000116	0.033128
ENSG00000007216	*SLC13A2*	-0.89	1.62E-05	0.01036
ENSG00000283413		-1.01	6.13E-05	0.02152
ENSG00000149021	*SCGB1A1*	-1.04	2.70E-05	0.013418
ENSG00000166106	*ADAMTS15*	-1.05	3.82E-08	0.00022
ENSG00000081052	*COL4A4*	-1.07	5.60E-05	0.020911
ENSG00000205502	*C2CD4B*	-1.1	3.62E-06	0.004526
ENSG00000099994	*SUSD2*	-1.2	7.28E-06	0.007098
ENSG00000198515	*CNGA1*	-1.22	4.47E-05	0.018715
ENSG00000289547		-1.22	1.39E-05	0.009656
ENSG00000274611	*TBC1D3*	-1.37	0.000178	0.039078
ENSG00000198787	*OR7E103P*	-1.52	1.42E-05	0.009656
ENSG00000113389	*NPR3*	-1.55	2.10E-06	0.003019
ENSG00000077522	*ACTN2*	-1.59	1.07E-05	0.008777
ENSG00000038295	*TLL1*	-1.63	8.21E-07	0.001487
ENSG00000174059	*CD34*	-1.83	5.43E-07	0.001354
ENSG00000250381	*UNC93B4*	-2.51	1.12E-08	0.000107
ENSG00000278599	*TBC1D3E*	-17.68	8.16E-08	0.000261

HGNC, HUGO Gene Nomenclature Committee; FC, fold change; FDR, False discovery rate.

Table S3. Significant correlations between differentially expressed EV miRNA and differentially expressed target genes epithelial brushings in patients with COPD

EV miRNA	Gene Symbol	Interaction type	R	FDR
**Upregulated miRNA**				
hsa-miR-223-3p	*CNGA1*	Predicted	-0.51	0.004
hsa-miR-223-3p	*ADAMTS15*	Predicted	-0.46	0.011
hsa-miR-2110	*PLAG1*	Validated	-0.44	0.015
hsa-miR-182-5p	*WNK4*	Validated	-0.43	0.016
hsa-miR-223-3p	*CYP24A1*	Predicted	-0.43	0.018
hsa-miR-223-3p	*INHBB*	Predicted	-0.40	0.033
hsa-miR-182-5p	*SCUBE3*	Predicted	-0.39	0.034
hsa-miR-2110	*PEG3*	Validated	-0.38	0.039
hsa-miR-2110	*COL4A4*	Predicted	-0.38	0.042
**Downregulated miRNA**				
hsa-miR-204-5p	*BDNF*	Validated	-0.54	0.002
hsa-miR-338-3p	*COL17A1*	Predicted	-0.46	0.01
hsa-miR-204-5p	*DSG3*	Predicted	-0.45	0.013
hsa-miR-204-5p	*TMEM132B*	Predicted	-0.43	0.017
hsa-miR-138-5p	*BDNF*	Predicted	-0.40	0.031
hsa-miR-338-3p	*CEMIP*	Predicted	-0.37	0.05

R - generated using Pearson's correlation coefficient. FDR, false discovery rate generated using the Benjamini-Hochberg method, with significance <0.05

Table S4. MicroRNA impact on the differential gene expression in epithelial brushings.

miRNA	Predicted genes identified	Percentage of gene targets	Percentage of DEGs
hsa-miR-182-5p	38	43.68	19.90
hsa-miR-138-5p	20	22.99	10.47
hsa-miR-204-5p	19	21.84	9.95
hsa-miR-338-3p	14	16.09	7.33
hsa-miR-2110	14	16.09	7.33
hsa-miR-223-3p	9	10.34	4.71
hsa-miR-200b-5p	5	5.75	2.62
hsa-miR-625-3p	2	2.30	1.05

DEGs, differentially expressed genes; miRNA, microRNA

Table S5. Significantly enriched GO Biological Process terms generated by BiNGO for the 87 unique mRNAs in the miRNA-mRNA interaction network.

GO ID	Description	Gene #	Mapped Genes	Cluster	p-value	FDR
**32868**	response to insulin stimulus	5	INSR|IRS2|INHBB|PDK1|TLR2	Metabolic Processes	0.00031	0.11
**7044**	cell-substrate junction assembly	3	COL17A1|ACTN2|PDK1	Immune cell regulation	0.00036	0.11
**48519**	negative regulation of biological process	21	KLF10|FGFBP1|ARG2|BDNF|INSR|JADE1|ARRB1|IRS2|INHBB|ARHGAP6|TOB1|CX3CL1|PER2|TAOK3|PSMD5|CILP|CLIP4|SACS|ATM|CD44|TLR2	System Processes	0.00038	0.11
**45428**	regulation of nitric oxide biosynthetic process	3	ARG2|INSR|TLR2	Response to Bacterial Lipopeptide	0.00044	0.11
**32869**	cellular response to insulin stimulus	4	INSR|IRS2|INHBB|PDK1	Metabolic Processes	0.00047	0.11
**71375**	cellular response to peptide hormone stimulus	4	INSR|IRS2|INHBB|PDK1	Metabolic Processes	0.00068	0.11
**43410**	positive regulation of MAPKKK cascade	4	TAOK3|INSR|ARRB1|CD44	Regulation of Cell Signaling	0.00068	0.11
**7160**	cell-matrix adhesion	4	COL17A1|ACTN2|CD44|PDK1	Immune cell regulation	0.00075	0.11
**44003**	modification by symbiont of host morphology or physiology	2	INSR|TLR2	Response to Bacterial Lipopeptide	0.00081	0.11
**32148**	activation of protein kinase B activity	2	INSR|PDK1	Metabolic Processes	0.00101	0.11
**45725**	positive regulation of glycogen biosynthetic process	2	INSR|IRS2	Metabolic Processes	0.00101	0.11
**70875**	positive regulation of glycogen metabolic process	2	INSR|IRS2	Metabolic Processes	0.00123	0.11
**43516**	regulation of DNA damage response, signal transduction by p53 class mediator	2	ATM|CD44	Cell Junction Organisation	0.00123	0.11
**9605**	response to external stimulus	9	CCL22|CYP24A1|INSR|ARRB1|INHBB|BMAL2|CX3CL1|CD44|TLR2	Cell Adhesion	0.00126	0.11
**31589**	cell-substrate adhesion	4	COL17A1|ACTN2|CD44|PDK1	Immune cell regulation	0.00132	0.11
**42221**	response to chemical stimulus	16	KLF10|CCL22|HMGCS1|EPAS1|BDNF|INSR|IRS2|AK4|INHBB|SLC7A11|CX3CL1|SCNN1G|CYP24A1|CD44|PDK1|TLR2	Cell Adhesion	0.00135	0.11
**7588**	excretion	3	SCNN1G|NPR3|ATP6V1B1	Excretion	0.002	0.12
**43434**	response to peptide hormone stimulus	5	INSR|IRS2|INHBB|PDK1|TLR2	Metabolic Processes	0.00207	0.12
**44092**	negative regulation of molecular function	7	ARG2|PSMD5|INSR|NPR3|ARRB1|IRS2|PDK1	Metabolic Processes	0.00212	0.12
**34329**	cell junction assembly	3	COL17A1|ACTN2|PDK1	Immune cell regulation	0.00223	0.12
**48041**	focal adhesion assembly	2	ACTN2|PDK1	Immune cell regulation	0.00231	0.12
**48523**	negative regulation of cellular process	18	KLF10|FGFBP1|BDNF|INSR|JADE1|ARRB1|IRS2|INHBB|ARHGAP6|TOB1|CX3CL1|PER2|TAOK3|PSMD5|CILP|CLIP4|ATM|CD44	System Processes	0.00231	0.12
**35468**	positive regulation of signaling pathway	7	FGFBP1|TAOK3|INSR|ARRB1|ATM|CD44|TLR2	Regulation of Cell Signaling	0.00231	0.12
**16477**	cell migration	6	SCNN1G|FERMT1|CCL22|CD34|CX3CL1|CD44	Cell Migration	0.00232	0.12
**10962**	regulation of glucan biosynthetic process	2	INSR|IRS2	Metabolic Processes	0.00263	0.12
**5979**	regulation of glycogen biosynthetic process	2	INSR|IRS2	Metabolic Processes	0.00263	0.12
**32885**	regulation of polysaccharide biosynthetic process	2	INSR|IRS2	Metabolic Processes	0.00263	0.12
**1817**	regulation of cytokine production	5	CLIP4|ARRB1|INHBB|CX3CL1|TLR2	Cell Adhesion	0.00289	0.12
**70873**	regulation of glycogen metabolic process	2	INSR|IRS2	Metabolic Processes	0.00298	0.12
**32881**	regulation of polysaccharide metabolic process	2	INSR|IRS2	Metabolic Processes	0.00298	0.12

GO ID	Description	Gene #	Mapped Genes	Cluster	p-value	FDR
**33280**	response to vitamin D	2	CYP24A1|INSR	Vitamin D Signaling	0.00298	0.12
**1657**	ureteric bud development	3	ARG2|BDNF|CD44	Ureteric Bud Development	0.00302	0.12
**70887**	cellular response to chemical stimulus	7	CYP24A1|EPAS1|INSR|IRS2|INHBB|CX3CL1|PDK1	Metabolic Processes	0.00304	0.12
**10646**	regulation of cell communication	13	FGFBP1|BDNF|INSR|ARRB1|IRS2|INHBB|TBC1D3|TAOK3|CILP|CLIP4|ATM|CD44|TLR2	System Processes	0.00314	0.12
**10907**	positive regulation of glucose metabolic process	2	INSR|IRS2	Metabolic Processes	0.00334	0.12
**10647**	positive regulation of cell communication	7	FGFBP1|TAOK3|INSR|ARRB1|ATM|CD44|TLR2	Regulation of Cell Signaling	0.00368	0.12
**48731**	system development	21	KLF10|COL17A1|EPAS1|PLAG1|BDNF|INSR|NPR3|FZD8|IRS2|INHBB|CX3CL1|SHROOM4|CYP24A1|COL4A1|COL4A4|ATOH8|FLNB|ATM|TLL1|CD44|ATP6V1B1	Developmental Processes	0.00397	0.12
**48870**	cell motility	6	SCNN1G|FERMT1|CCL22|CD34|CX3CL1|CD44	Cell Migration	0.00401	0.12
**51674**	localization of cell	6	SCNN1G|FERMT1|CCL22|CD34|CX3CL1|CD44	Cell Migration	0.00401	0.12
**45913**	positive regulation of carbohydrate metabolic process	2	INSR|IRS2	Metabolic Processes	0.00454	0.12
**10676**	positive regulation of cellular carbohydrate metabolic process	2	INSR|IRS2	Metabolic Processes	0.00454	0.12
**32677**	regulation of interleukin-8 production	2	ARRB1|TLR2	Response to Bacterial Lipopeptide	0.00454	0.12
**42892**	chloramphenicol transport	1	TLR2	Response to Bacterial Lipopeptide	0.00482	0.12
**50902**	leukocyte adhesive activation	1	CX3CL1	Developmental Processes	0.00482	0.12
**34238**	macrophage fusion	1	CD44	Cell Junction Organisation	0.00482	0.12
**48178**	negative regulation of hepatocyte growth factor biosynthetic process	1	INHBB	Hepatocyte Regulation	0.00482	0.12
**2158**	osteoclast proliferation	1	NPR3	Osteoclast Proliferation	0.00482	0.12
**51041**	positive regulation of calcium-independent cell-cell adhesion	1	CX3CL1	Developmental Processes	0.00482	0.12
**51040**	regulation of calcium-independent cell-cell adhesion	1	CX3CL1	Developmental Processes	0.00482	0.12
**48176**	regulation of hepatocyte growth factor biosynthetic process	1	INHBB	Hepatocyte Regulation	0.00482	0.12
**32646**	regulation of hepatocyte growth factor production	1	INHBB	Hepatocyte Regulation	0.00482	0.12
**15888**	thiamin transport	1	SLC19A2	Thiamine Transport	0.00482	0.12
**19087**	transformation of host cell by virus	1	INSR	Metabolic Processes	0.00482	0.12
**51817**	modification of morphology or physiology of other organism involved in symbiotic interaction	2	INSR|TLR2	Response to Bacterial Lipopeptide	0.00498	0.12
**34330**	cell junction organization	3	COL17A1|ACTN2|PDK1	Immune cell regulation	0.00505	0.12
**46326**	positive regulation of glucose import	2	INSR|IRS2	Metabolic Processes	0.00543	0.12
**48856**	anatomical structure development	22	KLF10|COL17A1|ARG2|EPAS1|PLAG1|BDNF|INSR|NPR3|FZD8|IRS2|INHBB|CX3CL1|SHROOM4|CYP24A1|COL4A1|COL4A4|ATOH8|FLNB|ATM|TLL1|CD44|ATP6V1B1	Developmental Processes	0.00547	0.12
**30036**	actin cytoskeleton organization	5	ARRB1|FLNB|ARHGAP6|SHROOM4|PDK1	Actin Organisation	0.00549	0.12

GO ID	Description	Gene #	Mapped Genes	Cluster	p-value	FDR
**10828**	positive regulation of glucose transport	2	INSR|IRS2	Metabolic Processes	0.00591	0.13
**7015**	actin filament organization	3	ARRB1|ARHGAP6|SHROOM4	Actin Organisation	0.00609	0.13
**43967**	histone H4 acetylation	2	JADE1|ARRB1	Histone Acetylation	0.0064	0.13
**45429**	positive regulation of nitric oxide biosynthetic process	2	INSR|TLR2	Response to Bacterial Lipopeptide	0.0064	0.13
**43255**	regulation of carbohydrate biosynthetic process	2	INSR|IRS2	Metabolic Processes	0.0064	0.13
**46777**	protein amino acid autophosphorylation	3	TAOK3|INSR|ATM	Regulation of Cell Signaling	0.00654	0.13
**30029**	actin filament-based process	5	ARRB1|FLNB|ARHGAP6|SHROOM4|PDK1	Actin Organisation	0.0071	0.14
**32870**	cellular response to hormone stimulus	4	INSR|IRS2|INHBB|PDK1	Metabolic Processes	0.00758	0.15
**43408**	regulation of MAPKKK cascade	4	TAOK3|INSR|ARRB1|CD44	Regulation of Cell Signaling	0.00758	0.15
**6928**	cellular component movement	7	SCNN1G|FERMT1|CCL22|BDNF|CD34|CX3CL1|CD44	Cell Migration	0.00771	0.15
**35467**	negative regulation of signaling pathway	5	TAOK3|CILP|CLIP4|ARRB1|CD44	Regulation of Cell Signaling	0.0086	0.15
**71495**	cellular response to endogenous stimulus	4	INSR|IRS2|INHBB|PDK1	Metabolic Processes	0.00896	0.15
**9719**	response to endogenous stimulus	7	HMGCS1|INSR|IRS2|AK4|INHBB|PDK1|TLR2	Metabolic Processes	0.00946	0.15
**7412**	axon target recognition	1	BDNF	Axon	0.00962	0.15
**2752**	cell surface pattern recognition receptor signaling pathway	1	TLR2	Response to Bacterial Lipopeptide	0.00962	0.15
**71726**	cellular response to diacylated bacterial lipopeptide	1	TLR2	Response to Bacterial Lipopeptide	0.00962	0.15
**71480**	cellular response to gamma radiation	1	ATM	Cell Junction Organisation	0.00962	0.15
**71727**	cellular response to triacylated bacterial lipopeptide	1	TLR2	Response to Bacterial Lipopeptide	0.00962	0.15
**42496**	detection of diacylated bacterial lipopeptide	1	TLR2	Response to Bacterial Lipopeptide	0.00962	0.15
**42495**	detection of triacylated bacterial lipopeptide	1	TLR2	Response to Bacterial Lipopeptide	0.00962	0.15
**32741**	positive regulation of interleukin-18 production	1	TLR2	Response to Bacterial Lipopeptide	0.00962	0.15
**33031**	positive regulation of neutrophil apoptosis	1	CD44	Cell Junction Organisation	0.00962	0.15
**60279**	positive regulation of ovulation	1	INHBB	Hepatocyte Regulation	0.00962	0.15
**33029**	regulation of neutrophil apoptosis	1	CD44	Cell Junction Organisation	0.00962	0.15
**60278**	regulation of ovulation	1	INHBB	Hepatocyte Regulation	0.00962	0.15
**71724**	response to diacylated bacterial lipopeptide	1	TLR2	Response to Bacterial Lipopeptide	0.00962	0.15
**71725**	response to triacylated bacterial lipopeptide	1	TLR2	Response to Bacterial Lipopeptide	0.00962	0.15
**70561**	vitamin D receptor signaling pathway	1	CYP24A1	Vitamin D Signaling	0.00962	0.15
**46324**	regulation of glucose import	2	INSR|IRS2	Metabolic Processes	0.00974	0.15

Terms were considered enriched using p-value <0.01. Cluster column indicates the name of the cluster each GO term was assigned to using AutoAnnotate, as visualised in Figure 4.

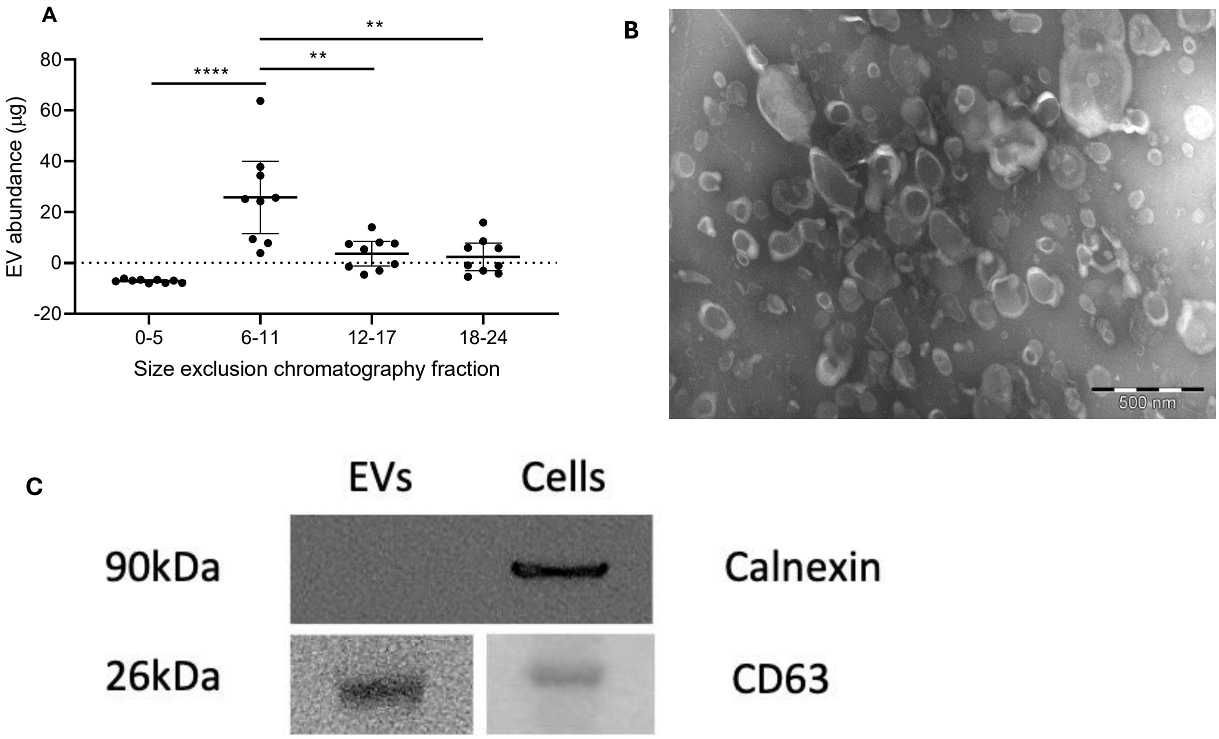

Figure S1. Characterisation of extracellular vesicles isolated from bronchoalveolar lavage fluid, n=9.

(A) EV abundance according to presence of CD9 in combined SEC fractions, with fractions 6-11 corresponding to EV containing fraction. **(B)** Whole mounted lung-derived EVs isolated from fractions 6-11 using SEC viewed by transmission electron microscopy. White arrow points to a characteristic EV with cup-shaped morphology and size between 30 – 150 nm. Scale bar shown in bottom right-hand corner. **(C)** Western blot analysis of isolated BAL EVs. 30ug of protein was used for Western blot analysis. Images captured from different WB for CD63 due to issues with running gel non-reducing conditions.

BAL, bronchoalveolar lavage; EV, extracellular vesicles; SEC, Size exclusion chromatography.

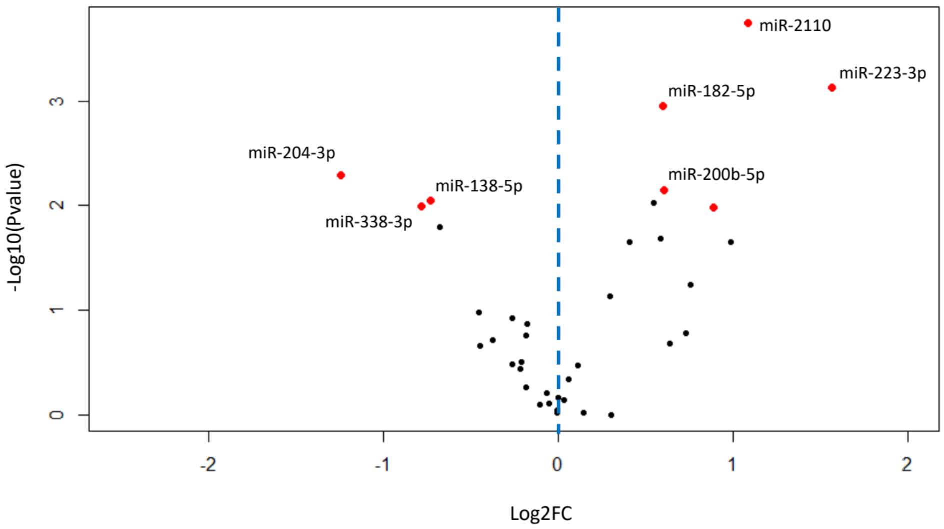

Figure S2. Volcano plot showing relationship between P values and expression data for differentially expressed miRNA validated by RT-qPCR.

Red dots show miRNA with P values <0.05 after FDR correction for multiple testing. Blue dotted line represents zero Log2FC, data points to the right are up-regulated in COPD, and data points to the left are down-regulated in COPD.

FC, fold change; FDR, false discovery rate; miRNA, microRNA; RT-qPCR, real-time quantitative PCR

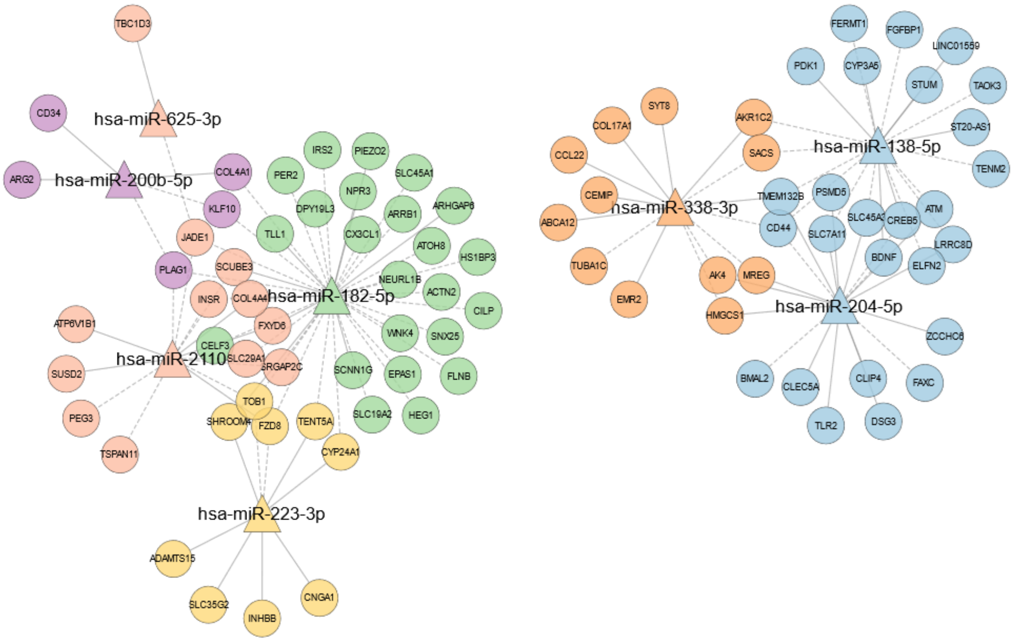

Cluster	miRNA	Regulation	Nodes #	In-degree	Modularity	Out-degree
**Cluster 1**	miR-204-5p, miR-138-5p	Down	28	34	4.857	7
**Cluster 2**	miR-182-5p	Up	24	23	1.533	15
**Cluster 3**	miR-2110, miR-625-3p	Up	14	13	1.3	10
**Cluster 4**	miR-338-3p	Down	13	12	1.714	7
**Cluster 5**	miR-223-3p	Up	10	9	1.286	7
**Cluster 6**	miR-200b-5p	Up	6	5	1.25	4

Figure S3. Enrichment map clusters with miRNA-specific interactions in gene sets.

Cluster analysis of the miRNA-mRNA network was performed using ClusterViz (v1.0.3), which identified six subclusters within the network. Triangular nodes represent differentially expressed EV miRNAs, while circular nodes represent DEGs in epithelial brushings. Each colour corresponds to a specific cluster: Cluster 1 (blue), Cluster 2 (green), Cluster 3 (red), Cluster 4 (orange), Cluster 5 (yellow), and Cluster 6 (purple).

DEGs, differentially expressed genes; EV, extracellular vesicle; miRNA, microRNA.

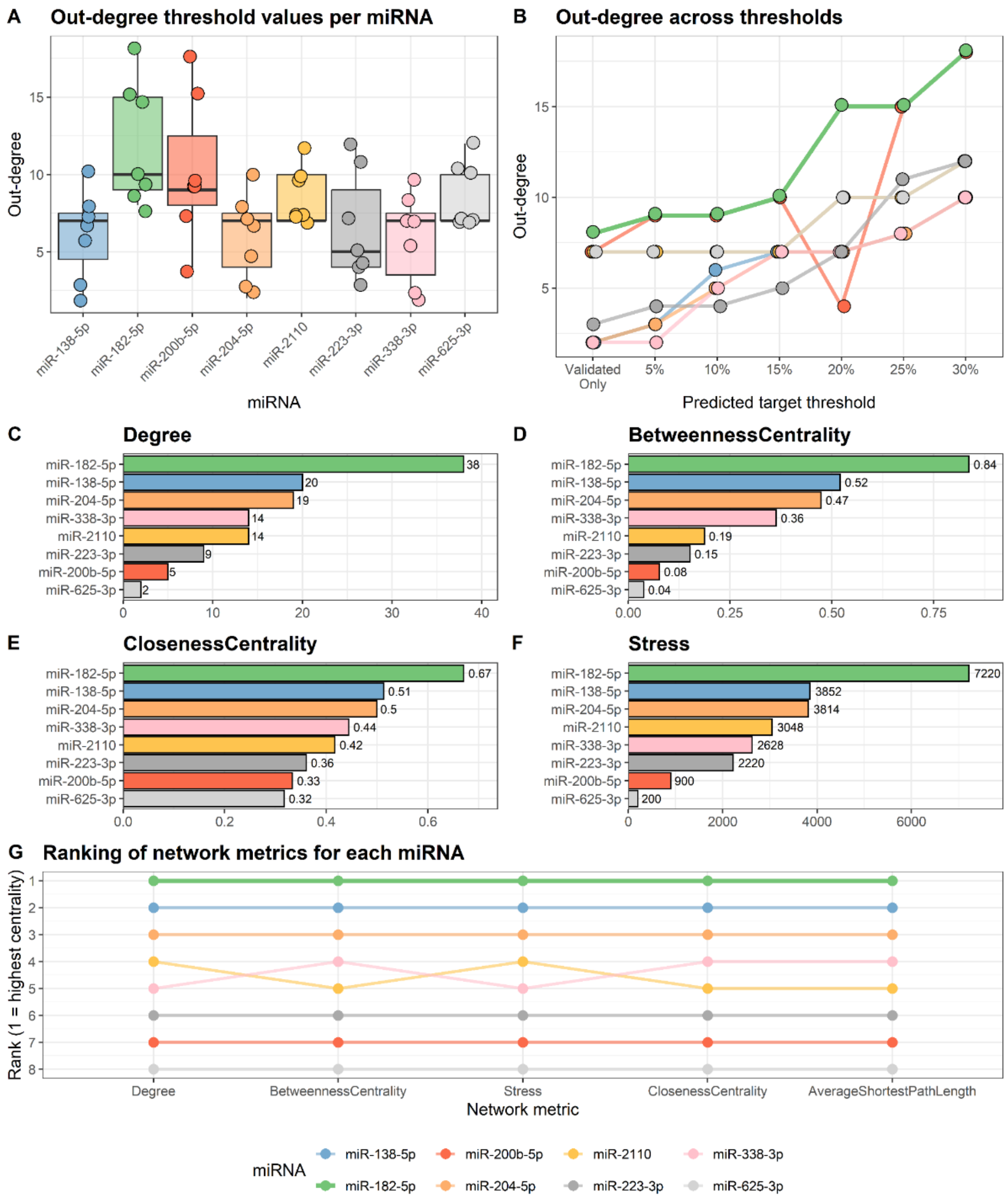

Figure S4. Assessment of miRNA network metrics across validated and predicted target thresholds.

Out-degree, calculated using *ClusterViz* (v1.0.3), is shown for each miRNA across experimentally validated targets and predicted targets at thresholds from 5-30%. **(A)** summarises the distribution of out-degree values per miRNA at each threshold, while **(B)** shows the out-degree of each miRNA at every threshold. Additional centrality metrics were also assessed at the chosen 20% predicted target threshold using the Network Analyser function in *Cytoscape* (v3.10.1). Panels **(C)-(F)** show bar plots of Degree, Betweenness Centrality, Closeness Centrality, and Stress, respectively, with bars ordered by descending value. **(G)** shows a rank plot summarising the relative position of each miRNA across all network metrics, highlighting the stability of these centrality rankings. Rank 1 corresponds to the highest centrality.

miRNA, microRNA

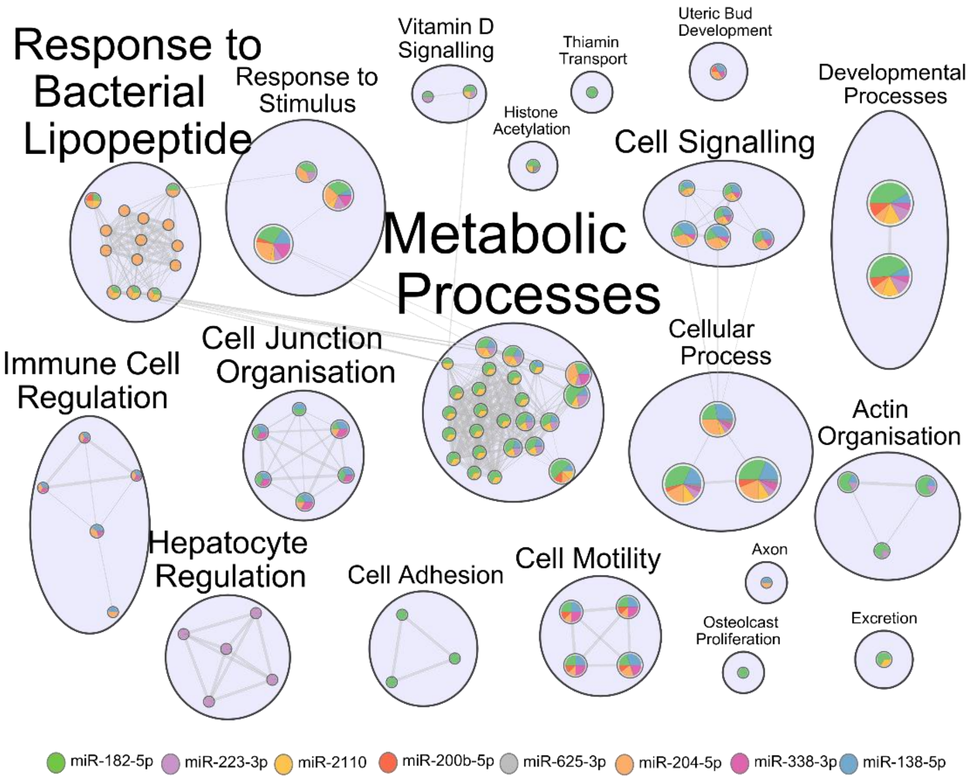

Figure S5. Enrichment map showing impact of miRNA on biological processes.

The nodes on the Enrichment map from Figure 4 were modified to visually represent the impact of each miRNA on each individual enriched GO term (node). Each node in the map contains a pie chart, with the proportion of the pie chart representing the percentage of genes out of the total number of genes assigned to a particular GO term that is targeted by a specific miRNA. The legend at the bottom of the plot indicates the colour assigned to each miRNA. The number of unique GO terms impacted by each miRNA: hsa-miR-182-5p = 66, hsa-miR-204-5p = 45, hsa-miR-2110 = 44, hsa-miR-138-5p = 42, hsa-miR-338-3p = 27, hsa-miR-223-3p = 26, hsa-miR-200b-5p = 12, hsa-miR-625-3p = 4.

GO; Gene Ontology; miRNA, microRNA.

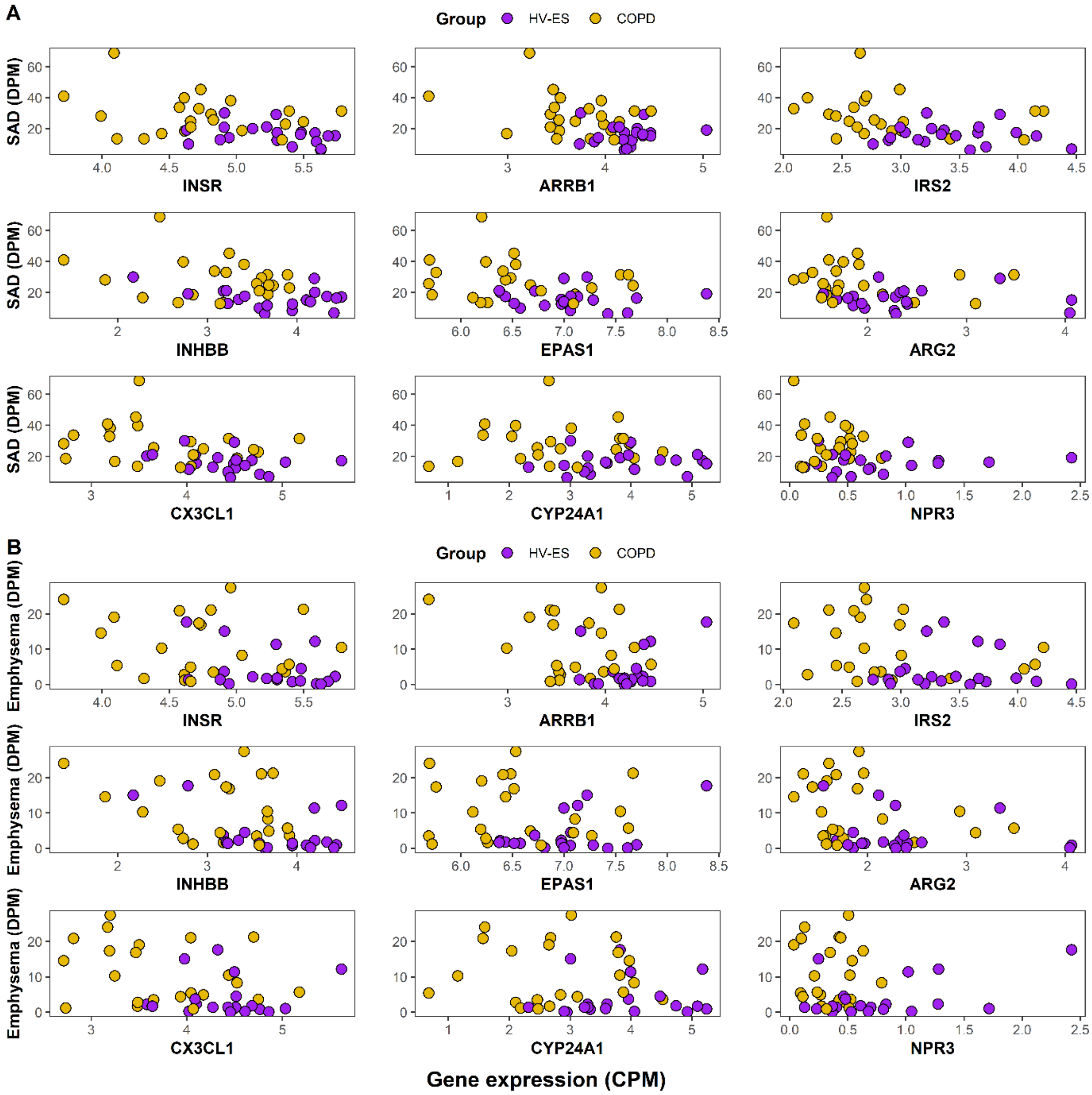

Figure S6. Scatter plots illustrating correlations of down-regulated DEGs with CT-derived DPM of (A) SAD and (B) Emphysema in COPD compared with HV-ES

COPD, Chronic Obstructive Pulmonary Disease; CT, computer tomography; DEGs, differentially expressed genes; DPM, disease probability measures; HV-ES, healthy volunteer ex-smokers; SAD, small airways disease
